# Characterization of a membrane binding loop leads to engineering botulinum neurotoxin B with improved therapeutic efficacy

**DOI:** 10.1371/journal.pbio.3000618

**Published:** 2020-03-17

**Authors:** Linxiang Yin, Geoffrey Masuyer, Sicai Zhang, Jie Zhang, Shin-Ichiro Miyashita, David Burgin, Laura Lovelock, Shu-Fen Coker, Tian-min Fu, Pål Stenmark, Min Dong

**Affiliations:** 1 Department of Urology, Boston Children’s Hospital, Department of Microbiology and Department of Surgery, Harvard Medical School, Boston, Massachusetts, United States of America; 2 Department of Biochemistry and Biophysics, Stockholm University, Stockholm, Sweden; 3 Ipsen Bioinnovation, Abingdon, United Kingdom; 4 Department of Biological Chemistry and Molecular Pharmacology, Harvard Medical School, Program in Cellular and Molecular Medicine, Boston Children’s Hospital, Boston, Massachusetts, United States of America; 5 Department of Experimental Medical Science, Lund University, Lund, Sweden; Institut Pasteur, FRANCE

## Abstract

Botulinum neurotoxins (BoNTs) are a family of bacterial toxins with seven major serotypes (BoNT/A–G). The ability of these toxins to target and bind to motor nerve terminals is a key factor determining their potency and efficacy. Among these toxins, BoNT/B is one of the two types approved for medical and cosmetic uses. Besides binding to well-established receptors, an extended loop in the C-terminal receptor-binding domain (H_C_) of BoNT/B (H_C_/B) has been proposed to also contribute to toxin binding to neurons by interacting with lipid membranes (termed lipid-binding loop [LBL]). Analogous loops exist in the H_C_s of BoNT/C, D, G, and a chimeric toxin DC. However, it has been challenging to detect and characterize binding of LBLs to lipid membranes. Here, using the nanodisc system and biolayer interferometry assays, we find that H_C_/DC, C, and G, but not H_C_/B and H_C_/D, are capable of binding to receptor-free lipids directly, with H_C_/DC having the highest level of binding. Mutagenesis studies demonstrate the critical role of consecutive aromatic residues at the tip of the LBL for binding of H_C_/DC to lipid membranes. Taking advantage of this insight, we then create a “gain-of-function” mutant H_C_/B by replacing two nonaromatic residues at the tip of its LBL with tryptophan. Cocrystallization studies confirm that these two tryptophan residues do not alter the structure of H_C_/B or the interactions with its receptors. Such a mutated H_C_/B gains the ability to bind receptor-free lipid membranes and shows enhanced binding to cultured neurons. Finally, full-length BoNT/B containing two tryptophan mutations in its LBL, together with two additional mutations (E1191M/S1199Y) that increase binding to human receptors, is produced and evaluated in mice in vivo using Digit Abduction Score assays. This mutant toxin shows enhanced efficacy in paralyzing local muscles at the injection site and lower systemic diffusion, thus extending both safety range and duration of paralysis compared with the control BoNT/B. These findings establish a mechanistic understanding of LBL–lipid interactions and create a modified BoNT/B with improved therapeutic efficacy.

## Abstract 2

Botulinum neurotoxins are a family of bacterial toxins, some of which are approved for medical and cosmetic uses. This study shows that introducing aromatic residues to a lipid binding loop improved therapeutic efficacy of botulinum neurotoxin B by enhancing its ability to bind to lipid membranes at motor nerve terminals.

## Introduction

Botulinum neurotoxins (BoNTs) are a family of protein toxins produced by various clostridial bacteria [1–3]. They target motor nerve terminals and block neurotransmitter release, resulting in muscle paralysis. These toxins are classified as a potential bioterrorism agent (category A and tier 1 select agent) in the United States [4], and members of BoNTs have also been used for treating medical conditions ranging from muscle spasm to chronic pain, as well as for cosmetic use in reducing wrinkles.

BoNTs are approximately 150-kDa proteins composed of a light chain (LC, approximately 50 kDa) and a heavy chain (HC, approximately 100 kDa), connected via a single disulfide bond [2, 3, 5, 6]. The HC includes the N-terminal translocation domain (H_N_, approximately 50 kDa) and the C-terminal receptor-binding domain (H_C_, approximately 50 kDa). BoNTs target motor nerve terminals by binding to specific receptors and enter neurons via receptor-mediated endocytosis. Acidification of endosomes triggers conformational changes in the toxin, leading to translocation of the LC across endosomal membranes into the cytosol, where the disulfide bond is reduced. The released LC acts as a zinc-dependent protease cleaving specific neuronal proteins.

BoNTs are traditionally classified into seven serotypes (BoNT/A–G) based on their distinct antigenicity. These serotypes may further contain a number of subtypes (e.g., BoNT/A1–A8) [7]. In addition, there are also chimeric toxins, such as BoNT/DC, which contains an LC-H_N_ derived from BoNT/D and an H_C_ most similar to BoNT/C-H_C_ (H_C_/C) [8–10]. Recent studies of clinical isolates also revealed a new mosaic toxin known as BoNT/H or BoNT/FA, with an LC most similar to a subtype of BoNT/F (BoNT/F5) and an H_C_ similar to BoNT/A1-H_C_ [11–13]. Most recently, a few BoNT-like toxins have been reported and characterized, including BoNT/X in a *Clostridium botulinum* strain [14], BoNT/En in an *Enterococcus faecium* strain [15], and Paraclostridial Mosquitocidal Protein 1 (PMP1) in *Paraclostridium bifermentans* strains [16].

BoNT/A, E, and C cleave the peripheral membrane protein synaptosomal nerve-associated protein 25 (SNAP-25) in neurons, and BoNT/B, D, F, and G cleave vesicle associated membrane protein (VAMP) 1, 2, and 3. BoNT/C can also cleave the plasma membrane protein Syntaxin 1. These three neuronal proteins belong to the soluble N-ethylmaleimide-sensitive factor (NSF) attachment protein receptor (SNARE) protein family and form a complex that is the core machinery mediating fusion of synaptic vesicle membranes to neuronal membranes [17, 18]. Cleavage of any one of the three SNARE proteins blocks synaptic vesicle exocytosis and neurotransmitter release.

A “double-receptor” model has been well established for the majority of BoNTs [19, 20]. As coreceptors, BoNTs utilize complex gangliosides (CGs), which are a family of glycolipids composed of a ceramide tail and various sialic acid–containing oligosaccharide headgroups [20]. A conserved ganglioside-binding site (GBS) with the core residues SxWY is conserved in BoNT/A, B, E, F, and G [21–28]. BoNT/DC is unique in that its GBS recognizes only the sialic acid moiety [29]. Besides gangliosides, BoNT/B, DC, and G utilize the synaptic vesicle membrane proteins synaptotagmin (Syt) I and II as their protein receptors [8, 30–34], whereas BoNT/A, E, and D utilize synaptic vesicle protein 2 (SV2), another family of synaptic vesicle membrane proteins [35–42]. BoNT/F was also reported to bind SV2, although the functional relevance of SV2 for BoNT/F remains to be established [39, 43–45].

Cocrystal structures of BoNT/B-Syt II and BoNT/DC-Syt I/II reveal that the Syt-binding site is close to but separate from the GBS [25, 46–49]. Both toxins recognize the same short amphipathic helix region adjacent to the single transmembrane domain of Syt I/II. The Syt-binding site in BoNT/G appears to be similar to that in BoNT/B [50, 51]. Binding to gangliosides and Syt I/II is not allosterically linked, as it does not induce any detectable conformational changes in BoNTs [25]. It was recently suggested that Syt I/II may interact with gangliosides and that both molecules together serve as the high-affinity receptors [52].

Among the seven BoNTs, BoNT/A and BoNT/B have been approved for clinical and cosmetic use, with BoNT/A as the dominant form in the market [53, 54]. In most applications, minute amounts of toxin are injected to reduce neuronal activity and relax the muscle surrounding the injection site. The therapeutic effects last months in humans, and patients can be injected repeatedly to maintain the effects over many years [54].

Enhancing toxin binding to nerve terminals facilitates their absorption into local neurons, thus reducing both diffusion from the injection site, which can lead to severe adverse effects [55], and the chance of triggering antibody responses, which renders future treatment ineffective [56]. For instance, BoNT/B shows lower efficacy in humans than BoNT/A, as a single residue change in human Syt II from the mouse version reduced binding affinity of BoNT/B to human Syt II [8, 48]. Thus, higher doses of BoNT/B are needed to achieve the same degree of muscle paralysis in humans compared with BoNT/A [57, 58], and clinical use of BoNT/B was associated with a higher frequency of adverse effects than BoNT/A [59, 60]. A structure-assisted mutagenesis screen identified a series of double point mutations, such as E1191M/S1199Y, within the Syt II-binding pocket of BoNT/B that can restore high-affinity binding to human Syt II [61]. This led to the creation of a mutant BoNT/B (e.g., BoNT/B^MY^) that can bind to human Syt II with high affinity. Whereas wild-type (WT) BoNT/B showed lower efficacy than BoNT/A in a humanized mouse model in which the Syt II luminal domain has been replaced with the human version, BoNT/B^MY^ and BoNT/A showed similar efficacy in paralyzing skeletal muscles in this humanized mouse model [62].

Interestingly, BoNT/B and BoNT/B^MY^ showed a higher potency than BoNT/A in paralyzing bladder muscles, which are smooth muscles [62]. This is consistent with clinical observations suggesting that BoNT/B may have advantages over BoNT/A in treating disorders involving smooth muscles and the autonomic nervous system [60, 63]. Thus, BoNT/B^MY^ may offer a higher therapeutic efficacy than BoNT/A for treating smooth muscle–related disorders [62].

When BoNT/B was modeled onto cell membranes via anchoring by gangliosides and Syt I/II, an extended loop between its GBS and Syt-binding site was recognized to be at a location ideal for membrane interactions [46, 47]. Similar loops exist in BoNT/C, DC, D, and G [10, 64]. Mutagenesis studies showed that mutations at the tip of this loop reduce binding of H_C_/C, H_C_/D, and H_C_/DC to immobilized gangliosides and cells, leading to the suggestion that this loop is involved in ganglioside binding [64–69]. Consistently, mutations at the tip of the loops in BoNT/C and BoNT/D reduced the potency of toxins in the mouse phrenic nerve hemidiaphragm assay (MPN) [65, 70]. However, there is no evidence that this loop specifically recognizes gangliosides. Instead, it can contribute to ganglioside binding by interacting with the hydrophobic membranes formed by the ceramide portion of gangliosides. Indeed, utilizing liposome flotation assays, we recently showed that H_C_/DC binds directly to liposomes containing only phosphatidylcholine (PC), and mutations at the tip of this extended loop abolish lipid-binding capability, demonstrating that this extended loop, designated “lipid-binding loop” (LBL), mediates membrane binding independent of gangliosides in BoNT/DC [29]. A recent study further examined the role of LBLs in BoNT/B, G, and DC, showing that deleting 3–5 residues in LBL drastically reduced the potency of BoNT/B, G, and DC [71]. Furthermore, H_C_/B, H_C_/G, and H_C_/DC with deletion mutations in their LBLs lost the ability to form stable binding to CGs or Syt II embedded in Triton X-100 micelles or in lipid environment in nanodiscs [71].

Here, we carried out a systematic characterization of LBL–lipid interactions in H_C_/B, C, D, G, and DC using the nanodisc system. We found that H_C_/C, DC, and G can bind to receptor-free lipid nanodiscs directly, whereas H_C_/B and D showed no detectable binding. Mutagenesis studies demonstrated the key role of having consecutive aromatic residues at the tip of LBL for binding of H_C_/DC to lipid membranes. A “gain-of-function” mutant H_C_/B was then created by replacing the two nonaromatic residues at the tip of the H_C_/B-LBL with tryptophan. Crystal structural studies confirmed that the two introduced tryptophan residues were located at the tip of H_C_/B-LBL and did not alter the conformation of H_C_/B. Such an engineered H_C_/B showed robust binding to receptor-free lipid membranes and enhanced binding to cultured neurons. Finally, we produced a full-length active form of BoNT/B containing the same two tryptophan mutations, in combination with the E1191M/S1199Y mutations (BoNT/B^MY-WW^), and found that this mutant toxin showed improved efficacy in paralyzing local muscles and reduced systemic diffusion, offering expanded safety ranges and longer therapeutic duration compared with BoNT/B^MY^.

## Results

### Characterizing binding of H_C_s to lipid membranes

**Fig 1:**
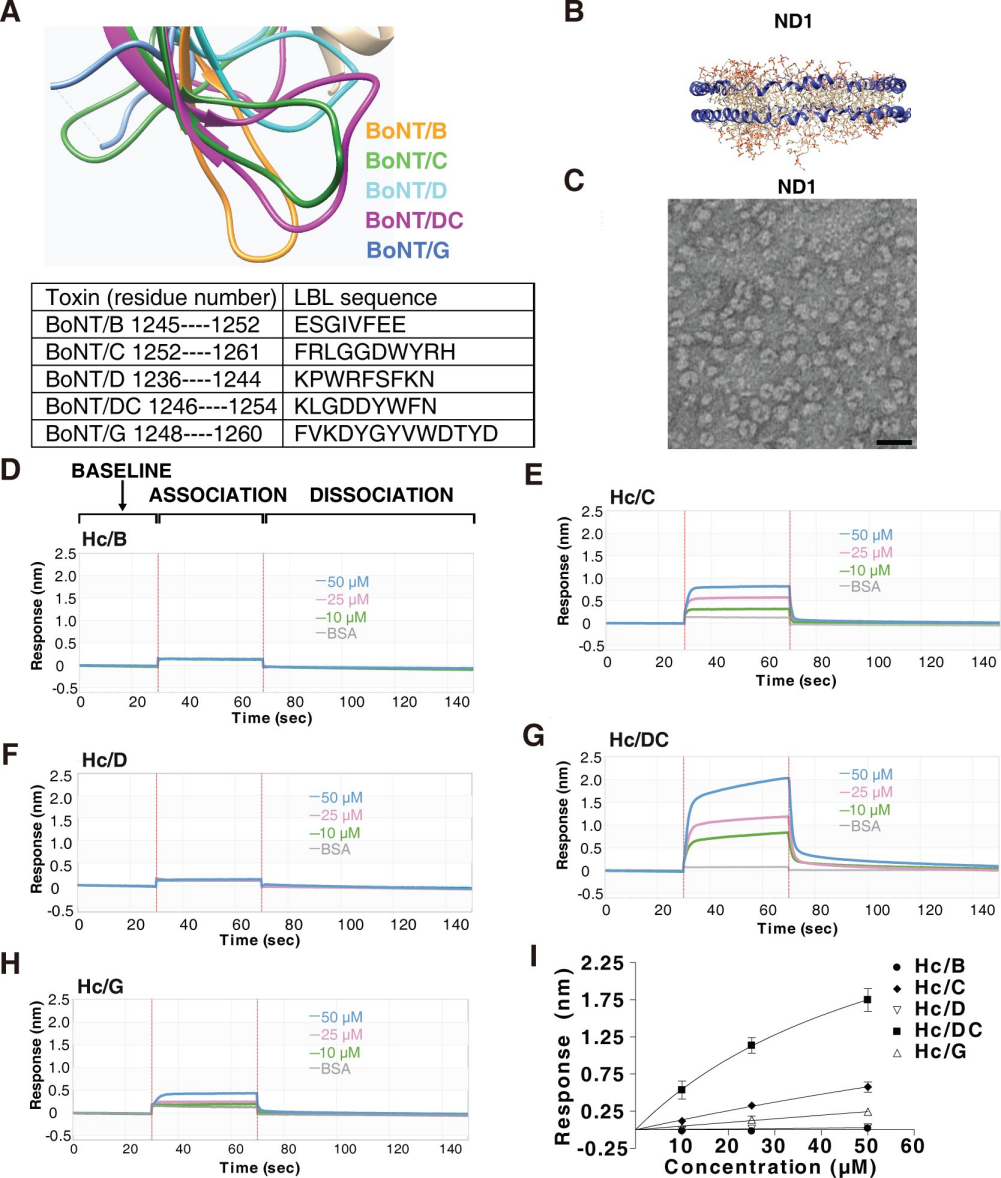
Characterizing binding of H_C_/B, /C, /D, /DC, and /G to lipid membranes in nanodiscs. (A) Structural overlay (top panel) and protein sequences (lower table) of the LBLs of H_C_/B, /C, /D, /DC, and /G. (B) Schematic drawing of nanodiscs (ND1). (C) The presence of nanodiscs was confirmed using negative staining EM. Scale bar represents 25 nm. (D–H). Binding of H_C_/B (panel D), H_C_/C (panel E), H_C_/D (panel F), H_C_/DC (panel G), and H_C_/G (panel H) to ND1 was examined using BLI assay. SA biosensors were exposed to three different concentrations of H_C_s (10, 25, and 50 μM) for 40 seconds (association phase), followed by an 80-second washing step (dissociation phase). Representative sensorgrams were color-coded (green: 10 μM; pink: 25 μM; blue: 50 μM) and shown, with the y-axis representing the shift in light wavelength upon binding (response nm). H_C_/B and H_C_/D did not show any detectable binding to ND1. H_C_/DC showed the strongest binding to ND1, and H_C_/C showed a modest level of binding, whereas H_C_/G showed low levels of binding. (I) The maximal binding signals of H_C_/B, /C, /D, /DC, and /G to ND1 as described in panels D–H were averaged and plotted (mean ± SD, *n* = 3). H_C_/DC showed the highest binding to ND1 among all H_C_s. Numerical values for (I) are available in S1 Data. BLI, biolayer interferometry; BSA, bovine serum albumin; EM, electron microscopy; H_C_, C-terminal receptor-binding domain; LBL, lipid-binding loop; ND1, receptor-free nanodisc; SA, streptavidin.

Among BoNTs, five (B, C, D, DC, and G) contain LBLs of considerable length (Fig 1A). To detect and characterize their direct binding to lipid membranes, we utilized nanodiscs, which are formed by circling lipid bilayers with amphipathic membrane scaffolding proteins (MSPs) such as Apolipoprotein A1 (ApoA1) (Fig 1B and S1A Fig) [72, 73]. Receptor-free nanodiscs (designated ND1) were generated using a mixture of three common cell membrane lipids: 1-palmitoyl-2-oleoyl-glycero-3-phosphocholine (POPC), 1,2-dioleoyl-sn-glycero-3-phospho-L-serine (DOPS), and cholesterol at the ratio of 74.5:5:20 [74]. They also contain a small percentage (0.5%) of biotin-PEG-DSPE (1,2-distearoyl-sn-glycero-3-phosphoethanolamine) in order to immobilize nanodiscs onto streptavidin (SA)-conjugated biosensors. The nanodiscs were purified by fast protein liquid chromatography (FPLC, S1A Fig), and negative staining electron microscopy (EM) confirmed the relative homogeneity of the nanodiscs (Fig 1C).

To monitor toxin binding to nanodiscs, we utilized the biolayer interferometry (BLI) assay, a label-free technology that offers real-time analysis of binding kinetics. Immobilization of biotinylated nanodiscs onto SA biosensors showed robust binding and virtually no dissociation (S1D Fig). As a control, nanodiscs that do not contain biotin-PEG-DSPE showed a low level of nonspecific background binding, which can be further reduced by including 0.5% bovine serum albumin (BSA) in the buffer (S1E Fig).

To analyze binding of recombinantly purified H_C_/B, H_C_/C, H_C_/D, H_C_/DC, and H_C_/G to immobilized ND1, SA biosensors loaded with ND1 were first immersed in the buffer containing BSA until a stable baseline was reached and then soaked in the buffer containing H_C_ for the association phase, followed by immersion in the buffer containing BSA again for the dissociation phase (Fig 1D). H_C_/B and H_C_/D showed no detectable binding to ND1 at concentrations of 10, 25, and 50 μM (Fig 1D and 1F), whereas H_C_/C and H_C_/DC showed binding to ND1 at all three concentrations (Fig 1E and 1G). H_C_/G showed low levels of binding at 25 and 50 μM (Fig 1H). These binding events are “fast-on” and “fast-off,” with binding affinity too low to be reliably quantified by the BLI assay. We instead compared the total binding levels at the end of the association phase. H_C_/DC showed the highest binding in a concentration-dependent manner, and H_C_/C showed lower levels of binding compared with H_C_/DC, whereas H_C_/G showed only low levels of binding (Fig 1I). Similar results were obtained using nanodiscs composed of only POPC (S2 Fig), demonstrating that the interactions are not dependent on the presence of DOPS or cholesterol. These data demonstrate direct binding of H_C_/DC, H_C_/C, and H_C_/G to receptor-free lipid membranes and suggest that LBLs are not created equal: H_C_/B and H_C_/D showed no detectable membrane interactions under our assay conditions.

### Consecutive aromatic residues in H_C_/DC-LBL are required for lipid binding

**Fig 2:**
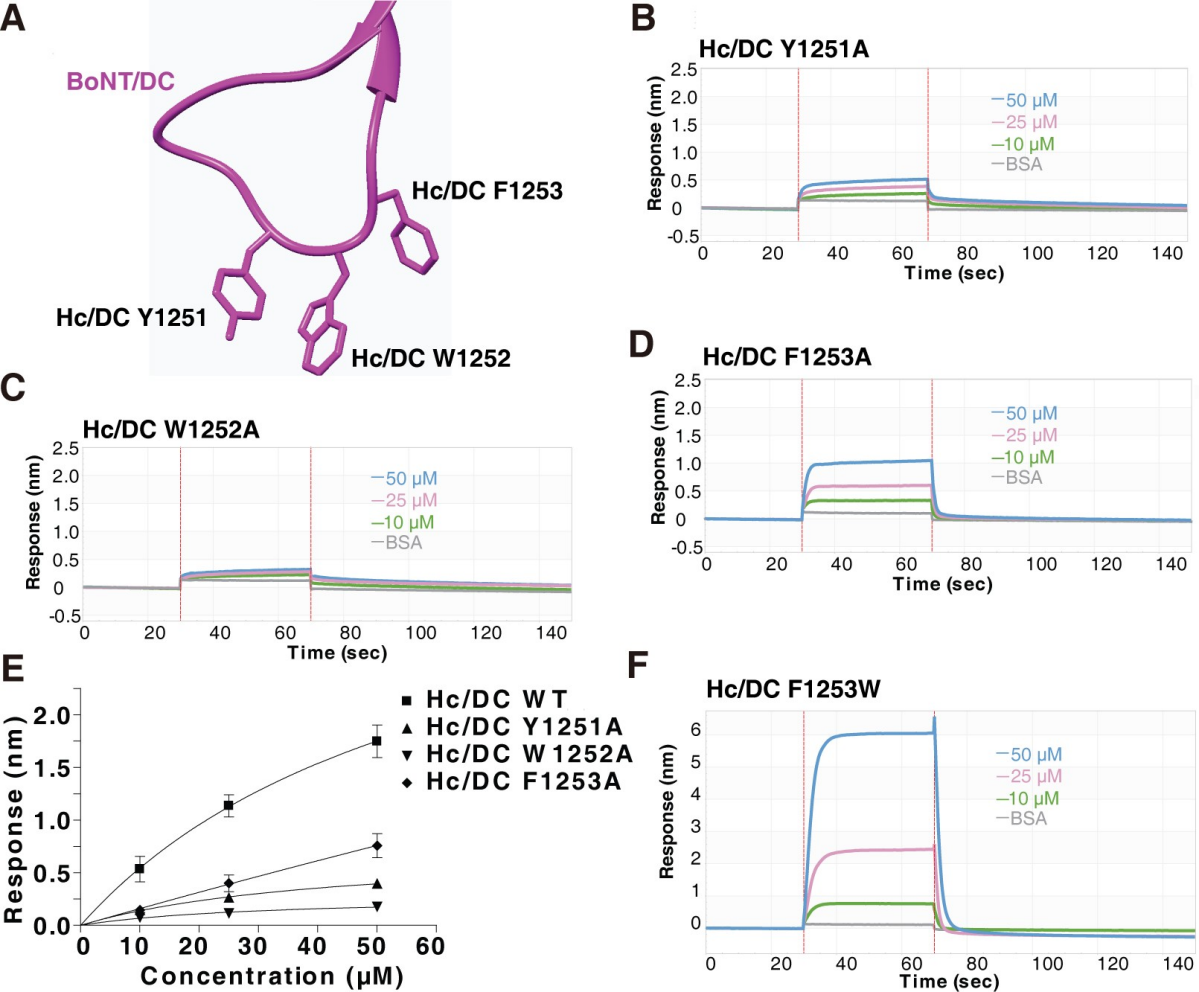
Mutating aromatic residues at the tip of H_C_/DC-LBL reduces its binding to lipid membranes. (A) Structure of the H_C_/DC-LBL showing the three aromatic residues at the tip. (B–D) Binding of H_C_/DC-LBL containing the indicated point mutations to ND1 was analyzed by BLI assays. Mutating the three aromatic residues at the tip of LBL reduced binding of H_C_/DC to ND1. (E) The maximal binding signals of H_C_/DC mutants as measured in panels B–D were averaged and plotted (mean ± SD, *n* = 3). Numerical values for (E) are available in S1 Data. (F) H_C_/DC-LBL (F1253W) showed robust binding to ND1. BLI, biolayer interferometry; BSA, bovine serum albumin; H_C_, C-terminal receptor-binding domain; LBL, lipid-binding loop; ND1, receptor-free nanodisc; WT, wild-type.

One difference among the five LBLs is the number of consecutive aromatic residues at the tip of the loop: H_C_/DC contains three (Y1251, W1252, and F1253, Figs 1A and 2A), and H_C_/C contains two (W1258, Y1259), whereas H_C_/B has only one (F1250). H_C_/D and H_C_/G both contain three aromatic residues, but they are not consecutive (Fig 1A). Aromatic residues such as Y, W, and F are ideal candidates to mediate membrane interactions, with W being the strongest [75]. We previously showed that mutating one of the three aromatic residues in H_C_/DC abolishes binding of H_C_/DC to liposomes in liposome flotation assays [29]. Consistently, point mutations Y1251A, W1252A, or F1253A in H_C_/DC all reduced total binding of H_C_/DC to ND1 in BLI assays (Fig 2B–2D), with F1253A showing the least and W1252A the strongest reduction (Fig 2E). In contrast, mutating F1253 to W increased total binding of H_C_/DC to ND1 (Fig 2F), serving as a gain-of-function mutation and demonstrating that aromatic residues at the tip of the LBL are critical for membrane interactions.

### H_C_/DC–lipid interactions synergize with Syt binding

It is well established that mutations at the three tip aromatic residues of H_C_/DC-LBL reduce H_C_/DC binding to gangliosides [29, 68, 69], suggesting that LBL–lipid interactions can contribute to overall binding affinity toward gangliosides on cell membranes. It remains unknown whether LBL–lipid interactions could play any meaningful roles in the presence of the protein receptor Syt II. To answer this question, we compared binding of H_C_/DC to free Syt II versus Syt II embedded in ND1 (designated ND-Syt, S1B and S1C Fig) using BLI assays. The recombinantly purified glutathione S-transferase (GST)-tagged Syt II luminal domain was immobilized onto biosensors and served as free Syt II. Binding of H_C_/DC to free Syt II was relatively weak, with an estimated apparent equilibrium dissociation constant (K_D_) of 4.8 × 10^−5^ M, whereas binding of H_C_/DC to ND-Syt showed an approximately 10-fold higher estimated K_D_ of 4.7 × 10^−6^ M (S3 Fig and Table 1). Mutating one of the three tip aromatic residues (Y1251A, W1252A, or F1253A) reduced the binding affinity of H_C_/DC to ND-Syt to a range of 1.9–4.8 × 10^−5^ M, similar to H_C_/DC binding to free Syt II (S3A Fig and Table 1). In contrast to these mutations that disrupt lipid binding, F1253W showed estimated K_D_ of 4.5 × 10^−6^ M for ND-Syt and 3.7 × 10^−5^ M for free Syt II, similar to WT H_C_/DC (S3A Fig and Table 1). Binding of these mutants to free Syt II showed estimated K_D_s within 2.7–7.5 × 10^−5^ M, similar to the K_D_ of WT H_C_/DC toward free Syt II (S3B Fig and Table 1). Thus, the reduction in their K_D_ to ND-Syt is likely due to loss of LBL–lipid interactions. These data suggest that LBL–lipid interactions increased overall binding affinity approximately 10-fold for H_C_/DC toward Syt II embedded in lipid membranes.

**Table 1:** Kinectics and affinity analysis of H_C_/DC and the indicated mutants to ND-Syt or free Syt II (1–61).

Ligand	Analyte	K_D_ (M)	ka (1/Ms)	ka Error	kd (1/s)	kd Error
**ND-Syt**	H_C_/DC WT	4.7E−06	2.5E+04	1.7E+03	1.2E−01	2.7E−03
	H_C_/DC Y1251A	3.3E−05	9.1E+03	4.0E+03	3.0E−01	1.7E−02
	H_C_/DC W1252A	4.8E−05	3.2E+03	3.7E+03	1.6E−01	1.4E−02
	H_C_/DC F1253A	1.9E−05	1.4E+04	2.2E+03	2.6E−01	8.9E−03
	H_C_/DC F1253W	4.5E−06	2.7E+04	1.4E+03	1.2E−01	1.6E−03
**Syt II (1–61)**	H_C_/DC WT	4.8E−05	6.4E+03	1.0E+03	3.1E−01	1.3E−02
	H_C_/DC Y1251A	7.5E−05	5.5E+03	1.7E+03	4.1E−01	2.7E−02
	H_C_/DC W1252A	2.6E−05	1.5E+04	5.3E+03	4.0E−01	4.0E−02
	H_C_/DC F1253A	2.7E−05	1.3E+04	2.1E+03	3.5E−01	1.9E−02
	H_C_/DC F1253W	3.7E−05	8.0E+03	9.6E+02	3.0E−01	1.1E−02

Abbreviations: H_C_, C-terminal receptor-binding domain; ka, association rate constant; kd, dissociation rate constant; K_D_, equilibrium dissociation constant; ND, nanodisc; Syt, synaptotagmin; WT, wild-type

### Introducing tryptophan residues into H_C_/B-LBL

**Fig 3:**
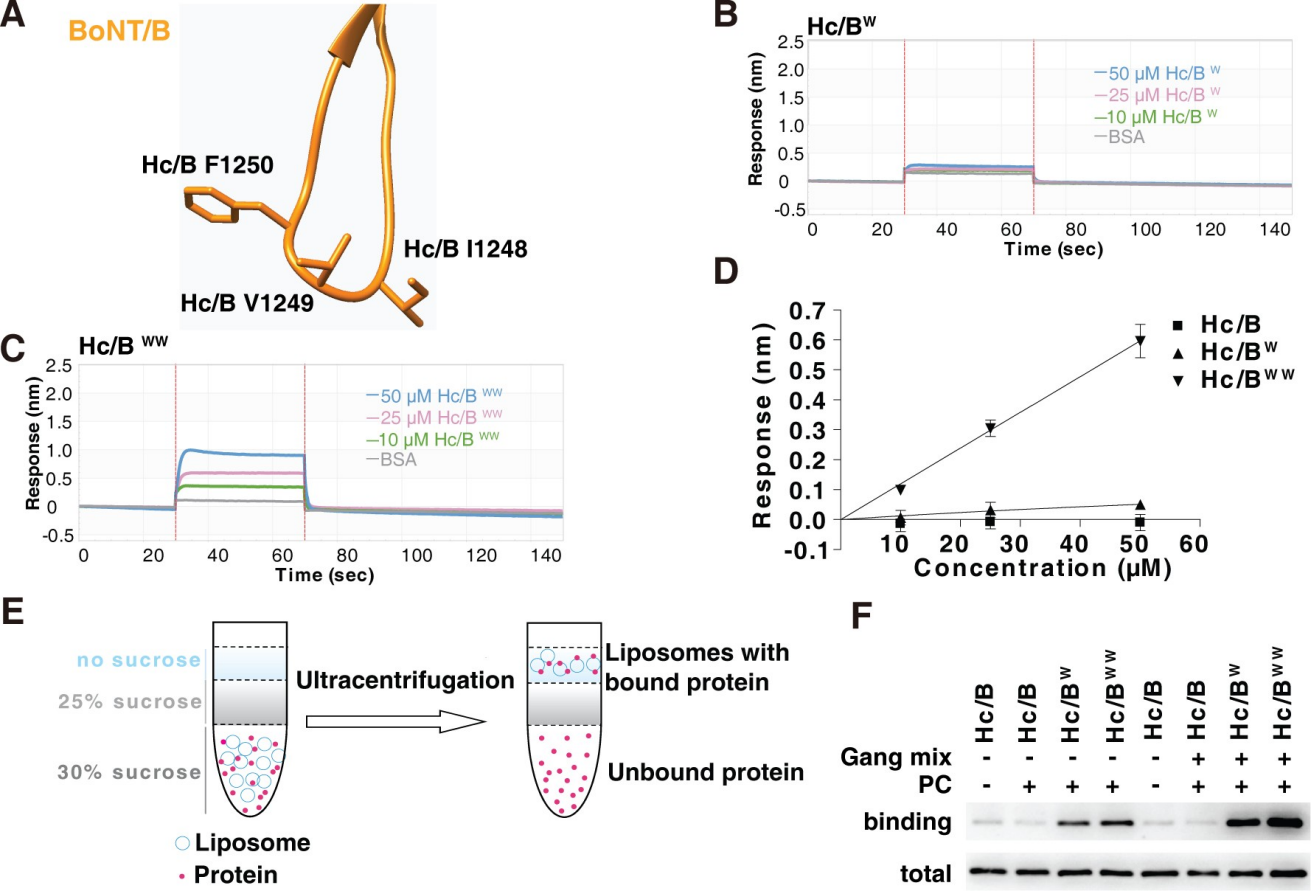
Introducing aromatic residues at the tip of H_C_B-LBL enables its binding to lipid membranes. (A) Structure of H_C_B-LBL, with three residues at the tip marked. (B–C) Binding of H_C_/B mutants containing either a single point mutation, I1248W, or double point mutations, I1248W/V1249W, to ND1 were analyzed by BLI assays. H_C_/B^W^ showed low-level binding to ND1, whereas H_C_/B^WW^ showed robust binding. (D) The maximal binding signals of H_C_/B mutants as measured in panels B and C were averaged and plotted (mean ± SD, *n* = 3). Numerical values for (D) are available in S1 Data. (E–F) Binding of WT H_C_/B, H_C_/B^W^, and H_C_/B^WW^ to liposomes containing POPC or POPC plus gangliosides were analyzed by liposome flotation assays. Proteins bound to liposomes floated to the top of the sucrose density gradient and were collected and subjected to immunoblot analysis. H_C_/B^W^ and H_C_/B^WW^ were able to bind POPC liposomes, and the presence of gangliosides further enhanced their binding. BLI, biolayer interferometry; BoNT, botulinum neurotoxin; BSA, bovine serum albumin; H_C_, C-terminal receptor-binding domain; LBL, lipid-binding loop; ND1, receptor-free nanodisc; PC, phosphatidylcholine; POPC, 1-palmitoyl-2-oleoyl-glycero-3-phosphocholine; WT, wild-type.

The tip of H_C_/B-LBL contains I1248, V1249, and a single aromatic residue, F1250 (Fig 3A). We next investigated whether introducing consecutive aromatic residues into H_C_/B-LBL creates gain-of-function mutations that enable H_C_/B-LBL to bind lipid membranes. We first attempted to replace the entire H_C_/B-LBL with H_C_/DC-LBL, but the resulting chimeric H_C_s did not show any binding to ND1 (S4 Fig). It is possible that the LBL in chimeric H_C_ has a folding/location different from the LBL in H_C_/DC. We next examined the effect of replacing the tip residues I and V with aromatic ones, including I1248W (designated H_C_/B^W^) and a double mutation (I1248W/V1249W, designated H_C_/B^WW^). H_C_/B^W^ showed a low level of binding to ND1, whereas H_C_/B^WW^ showed a much higher binding than H_C_/B^W^ (Fig 3B–3D). These bindings showed fast-on and fast-off kinetics, similar to H_C_/DC binding to ND1. We also attempted to add one more aromatic residue (S5 Fig). One triple mutation (G1247W/I1248Y/V1249W) showed higher levels of binding to ND1 than H_C_/B^WW^, but the protein had poor solubility.

To further confirm that H_C_/B^WW^ binds lipid membranes directly, we examined the association of WT H_C_/B, H_C_/B^W^, and H_C_/B^WW^ to liposomes in flotation assays. Liposomes containing only PC or PC plus gangliosides were incubated with H_C_s and then subjected to ultracentrifugation in sucrose gradients. Proteins bound to liposomes floated to the top of the gradient and were analyzed by immunoblot (Fig 3E) [29]. H_C_/B^W^ and H_C_/B^WW^ showed direct binding to liposomes containing only PC (Fig 3F). The presence of gangliosides further enhanced binding of H_C_/B^W^ and H_C_/B^WW^, whereas WT H_C_/B showed no binding to either PC liposomes or ganglioside-containing liposomes under our assay conditions.

### Lipid binding synergizes with H_C_/B–ganglioside interactions

**Fig 4:**
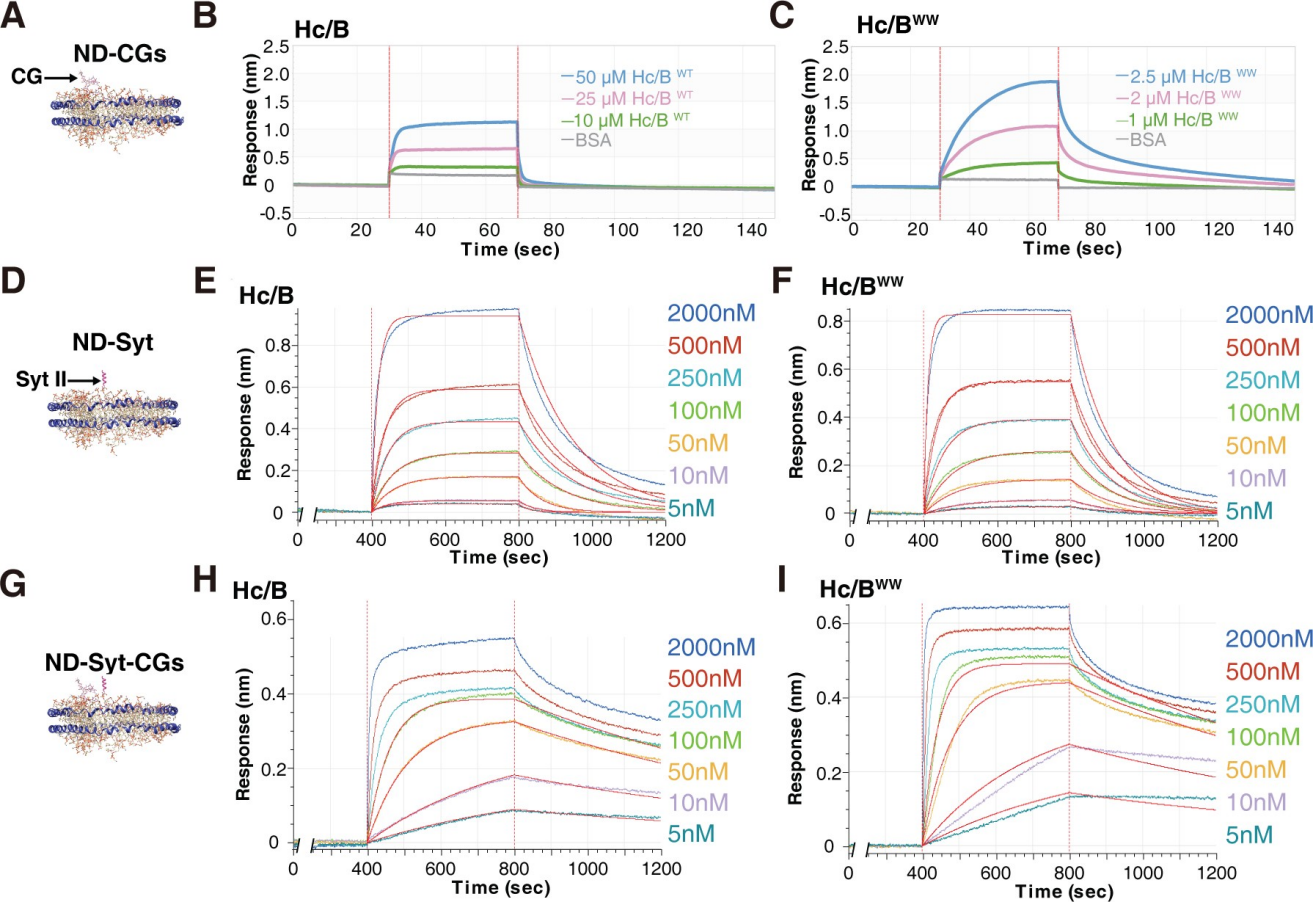
Lipid-binding capability synergizes with BoNT/B-CGs interactions. (A–C) Binding of WT H_C_/B and H_C_/B^WW^ to ND-CGs was analyzed by BLI assays. Binding kinetics were analyzed and listed in S1 Table. (D–F) Binding of WT H_C_/B and H_C_/B^WW^ to ND-Syt was analyzed by BLI assays using the Octet RED 384 system at the indicated concentrations. Binding curves were fitted using a 1:1 binding model, and fitted lines are shown in red. Binding kinetics were analyzed and listed in S1 Table. (G–I) Binding of WT H_C_/B and H_C_/B^WW^ to ND-Syt-CGs, which contains both Syt II and CGs, was analyzed by BLI assays using the Octet RED 384 system at the indicated concentrations. Interactions involve at least three heterogeneous components, and it is not possible to draw a precise binding K_D_ for comparison. Binding curves were fitted using a 1:1 binding model, and fitted lines are shown in red. Estimated K_D_ using a single–binding site model is included in S1 Table. BLI, biolayer interferometry; BoNT, botulinum neurotoxin; BSA, bovine serum albumin; CG, complex ganglioside; H_C_, C-terminal receptor-binding domain; K_D_, equilibrium dissociation constant; ND, nanodisc; Syt, synaptotagmin; WT, wild-type.

We next analyzed whether the novel lipid-binding capability may contribute to H_C_/B binding to gangliosides and/or Syt II within lipid membranes. To analyze the effect in the presence of gangliosides, new nanodiscs were generated by adding CGs to ND1, designated ND-CGs (Figs 4A and S1C–S1E, 1% disialoganglioside (GD1a) and 1% trisialoganglioside (GT1b), POPC is reduced proportionally to 72.5%). Although WT H_C_/B showed binding to ND-CGs only at high concentrations, H_C_/B^WW^ showed higher levels of binding already at much lower concentrations (Fig 4B and 4C). For instance, incubation with 2.5 μM H_C_/B^WW^ achieved a higher level of binding than incubation with 50 μM of WT H_C_/B. Analysis of estimated K_D_ revealed an approximately 5-fold increase: K_D_ approximately 1.2 × 10^−5^ M for H_C_/B^WW^ versus 5.1 × 10^−5^ M for WT H_C_/B (S1 Table).

To examine the effect of lipid binding in the presence of Syt II, we compared binding of H_C_/B versus H_C_/B^WW^ for ND-Syt. As binding of H_C_/B to Syt II has a relatively high affinity, with K_D_ approximately 10^−7^ M [46, 47, 71], we resorted to the more-sensitive Octet RED 384 system for BLI assays and titrated the H_C_ concentrations from 2 μM down to 5 nM (Fig 4D–4F). Binding responses, total binding levels, and dissociation rates were all similar between WT H_C_/B and H_C_/B^WW^. Both showed binding K_D_ at approximately 10^−7^ M levels for ND-Syt (S1 Table). These results suggest that binding to ND-Syt is dominated by the strong H_C_/B-Syt II interactions; the effect of lipid binding was negligible under our assay conditions.

To analyze the effect of lipid binding in the presence of both Syt II and CGs, we generated nanodiscs containing Syt II and CGs, designated ND-Syt-CGs (Fig 4G and S1C–S1E Fig). H_C_/B binding to these nanodiscs involves at least three heterogeneous components (LBL–lipid, H_C_/B–gangliosides, and H_C_/B-Syt II), and it is impractical to draw a binding K_D_ for comparison. Using a single–binding site model to fit the binding/dissociation curves suggests an estimated binding K_D_ at approximately 10^−9^ M levels for H_C_/B and H_C_/B^WW^ (S1 Table). Interestingly, H_C_/B^WW^ showed slightly greater total binding, earlier saturation, and slower dissociation for binding to ND-Syt-CGs compared with WT H_C_/B (Fig 4H and 4I), although the overall binding is likely dominated by H_C_/B-Syt II–ganglioside interactions.

### H_C_/B^WW^ showed enhanced binding to cultured neurons

**Fig 5:**
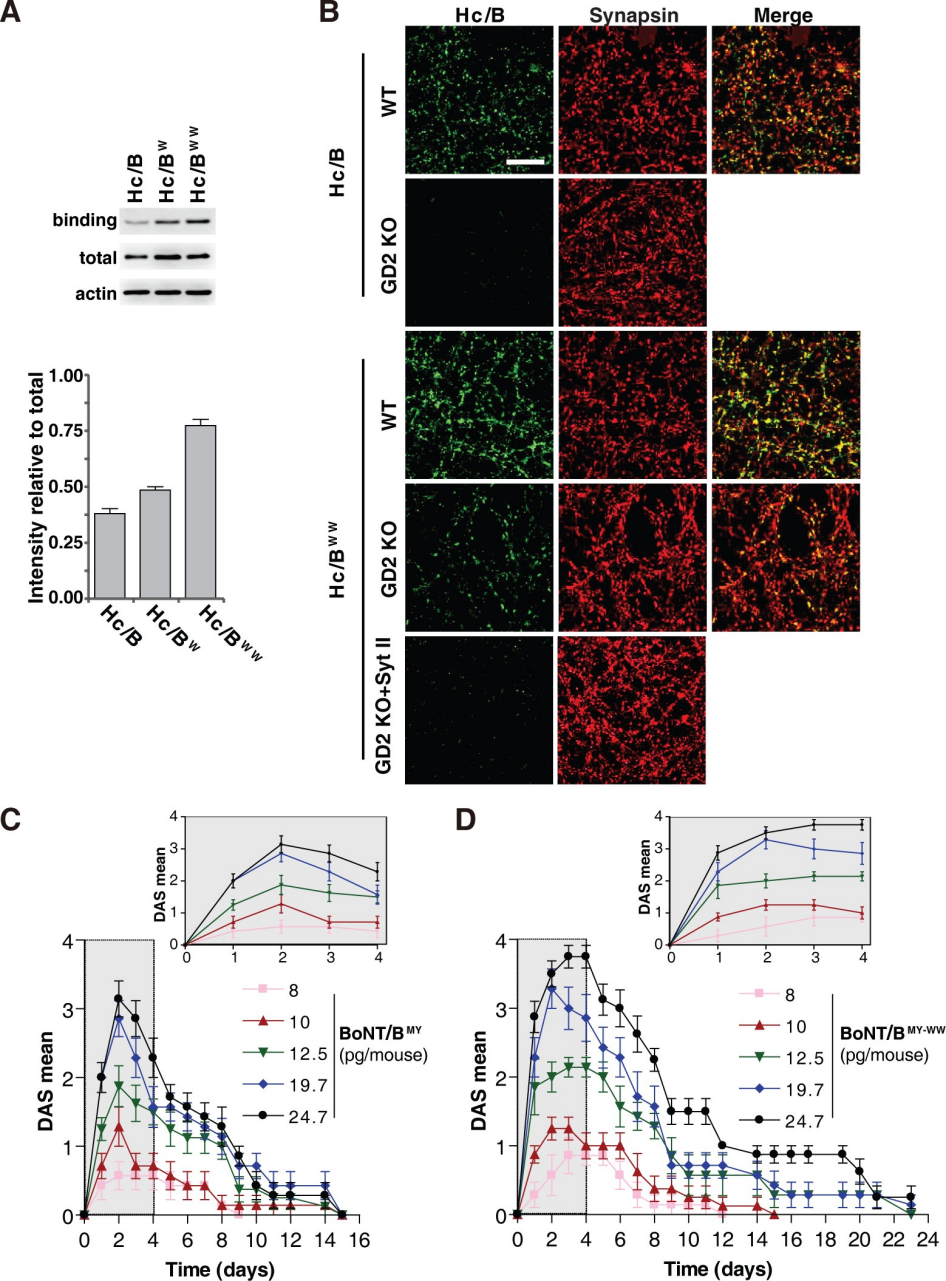
Characterizing binding of H_C_/B^WW^ on cultured neurons and BoNT/B^MY-WW^ in vivo. (A) Binding of WT H_C_/B, H_C_/B^W^, and H_C_/B^WW^ to cultured rat cortical neurons was examined by immunoblot analysis. Neurons were exposed to HA-tagged H_C_/B, H_C_/B^W^, and H_C_/B^WW^ for 5 minutes, washed, and harvested for immunoblot analysis. Actin served as an internal loading control. The total represents 50 ng of H_C_ proteins. Representative blots and the intensity relative to total H_C_/B quantification results are shown. (B) Binding of H_C_/B and H_C_/B^WW^ to cultured mouse cortical WT and GD2 KO neurons was examined by immunostaining analysis. Neurons were exposed to HA-tagged H_C_/B, H_C_/B^WW^, or H_C_/B^WW^ plus GST-tagged recombinant Syt II (1–61, 10-fold of H_C_/B^WW^ concentration) for 5 minutes, washed, fixed, and subjected to immunostaining analysis. Synapsin serves as an internal marker for presynaptic terminals. H_C_/B^WW^ showed enhanced binding to neurons compared with H_C_/B and colocalized with Synapsin. Binding of H_C_/B^WW^ is lower on GD2 KO neurons compared with WT neurons, and residual binding of H_C_/B^WW^ on GD2 KO neurons is eliminated by recombinant Syt II fragment protein as a receptor decoy. Scale bar represents 30 μm. (C) The control BoNT/B^MY^ elicited a dose-dependent response in the DAS mean score. Data are means ± SEM of *n* = 7 or 8. The response within the first 4 days is shown in the enlarged insert. (D) BoNT/B^MY-WW^ showed a more potent response and longer duration in the DAS assay than the control BoNT/B (panel C). Data are means ± SEM of *n* = 7 or 8. Numerical values for (A), (C), and (D) are available in S1 Data. BoNT, botulinum neurotoxin; DAS, Digit Abduction Score; GD2 KO, *Galgt1* knockout; GST, glutathione S-transferase; HA, human influenza hemagglutinin; H_C_, C-terminal receptor-binding domain; Syt, synaptotagmin; WT, wild-type.

We next analyzed whether lipid binding contributes to H_C_/B binding to neuronal surfaces. Cultured rat cortical neurons were incubated with WT H_C_/B, H_C_/B^W^, or H_C_/B^WW^. Cells were washed, and cell lysates were harvested. Bound H_C_s were detected via immunoblot analysis. H_C_/B^W^ showed slightly higher binding than H_C_/B, whereas H_C_/B^WW^ showed much higher total binding compared with H_C_/B (Fig 5A).

To further examine whether enhanced binding of H_C_/B^WW^ is due to nonspecific attachment to cell membranes, we carried out immunostaining assays on binding of H_C_/B^WW^ to both WT neurons and neurons cultured from knockout mice lacking the necessary enzyme to synthesize CGs (*Galgt1* knockout [GD2 KO]) [29]. Consistent with the immunoblot analysis results, H_C_/B^WW^ showed overall stronger immunostaining signals than H_C_/B on both WT neurons and GD2 KO neurons (Fig 5B). Binding of H_C_/B^WW^ is largely colocalized with the presynaptic terminal marker Synapsin on both WT and GD2 KO neurons (Fig 5B), indicating the specificity of H_C_/B^WW^ toward nerve terminals. Binding of H_C_/B^WW^ is much lower on GD2 KO neurons compared with WT neurons (Fig 5B). Furthermore, residual binding of H_C_/B^WW^ to GD2 KO neurons can be eliminated using a soluble recombinant protein of Syt II fragment as a receptor decoy (residues 1–61, Fig 5B). These data demonstrate that H_C_/B^WW^ binding to neurons still depends on both gangliosides and Syt I/II, which ensures its specificity toward nerve terminals.

### Characterizing BoNT/B^WW^ mutant toxin in vivo

We next sought to evaluate the impact of WW mutations on in vivo activity of BoNT/B. Full-length BoNT/B containing the WW mutations in its LBL (BoNT/B^MY-WW^) and control BoNT/B were produced recombinantly in *Escherichia coli*. Both toxins also contained the same set of additional mutations (E1191M/S1199Y, BoNT/B^MY^) that we previously developed for enhancing binding to human Syt II [61]. They were produced and activated in the same way, and the only differences were the WW mutations. We compared the potential therapeutic efficacy of these two toxins using a well-established local paralysis assay known as the Digit Abduction Score (DAS) assay [76, 77]. Injecting sublethal levels of toxins into the leg muscle paralyzes it and prevents spreading of the toes. The degree of paralysis is scored 0–4 (4 representing complete paralysis, Fig 5C and 5D). The median effective dose (ED50) represents the toxin dose that induces score-2 level of paralysis. The 0% body weight change (%BW) represents the toxin dose that does not result in any reduction in body weight, in turn reflecting the degree of systemic diffusion of the toxin from the injection site. The ratio between these two doses represents the safety range for the toxin, a key pharmacological parameter. BoNT/B^MY-WW^ showed a slightly better ED50 than the control BoNT/B^MY^, although the difference was not significant (Table 2). The major difference was 0%BW for BoNT/B^MY-WW^: up to approximately 23 pg can be injected without causing body weight reduction, whereas only approximately 15 pg of BoNT/B^MY^ can be injected. Thus, the safety ratio for BoNT/B^MY-WW^ was almost twice that of BoNT/B^MY^ (1.94 versus 0.99, Table 2). Furthermore, BoNT/B^MY-WW^ at the safe dose of 19.7 pg induced paralysis that lasted approximately 23 days (Fig 5D), much longer than the same dose of BoNT/B^MY^ (approximately 15 days, Fig 5C).

**Table 2:** Mean ED50, 0%BW, and LD50 following IM injection in mice.

Toxin	ED50 (pg/mouse)	0%BW (pg/mouse)	0%BW/ED50	Estimated LD50 (pg/mouse)
**BoNT/B^MY^**	14.2 ± 2.0	14.7 ± 2.8	0.99 ± 0.02	29.0 ± 4.3
**BoNT/B^MY-WW^**	11.8 ± 0.6	23.0 ± 3.4	1.94 ± 0.38	35.4 ± 1.0

Values are shown as means ± SD of *n* = 7 or 8 independent experiments performed in triplicate.

Abbreviations: BoNT, botulinum neurotoxin; BW, body weight change; ED50, median effective dose; IM, intramuscular; LD50, median lethal dose

These improvements are consistent with our findings that BoNT/B^MY-WW^ has enhanced binding to neuronal synapses, allowing it to be absorbed by local neurons more efficiently than the control toxin. It is also possible that the ability of BoNT/B^MY-WW^ to bind lipids with fast-on/fast-off low affinity can slow the overall diffusion of the toxin within local tissues, increasing its chances of encountering local nerve terminal receptors.

Finally, we also estimated the systemic toxicity levels of BoNT/B^MY-WW^ in mice via intramuscular injection (intramuscular median lethal dose [IM LD50]). A series of toxin dilutions were injected into the hind legs of mice. Survival rates were monitored, and IM LD50s were estimated at 29.0 ± 4.3 pg for the control BoNT/B and 35.4 ± 1.0 pg for BoNT/B^MY-WW^ (Table 2). These data suggest that BoNT/B^MY-WW^ has a slightly lower systemic toxicity than BoNT/B^MY^.

### Crystal structure of H_C_/B^WW^ in complex with GD1a and Syt I

**Fig 6:**
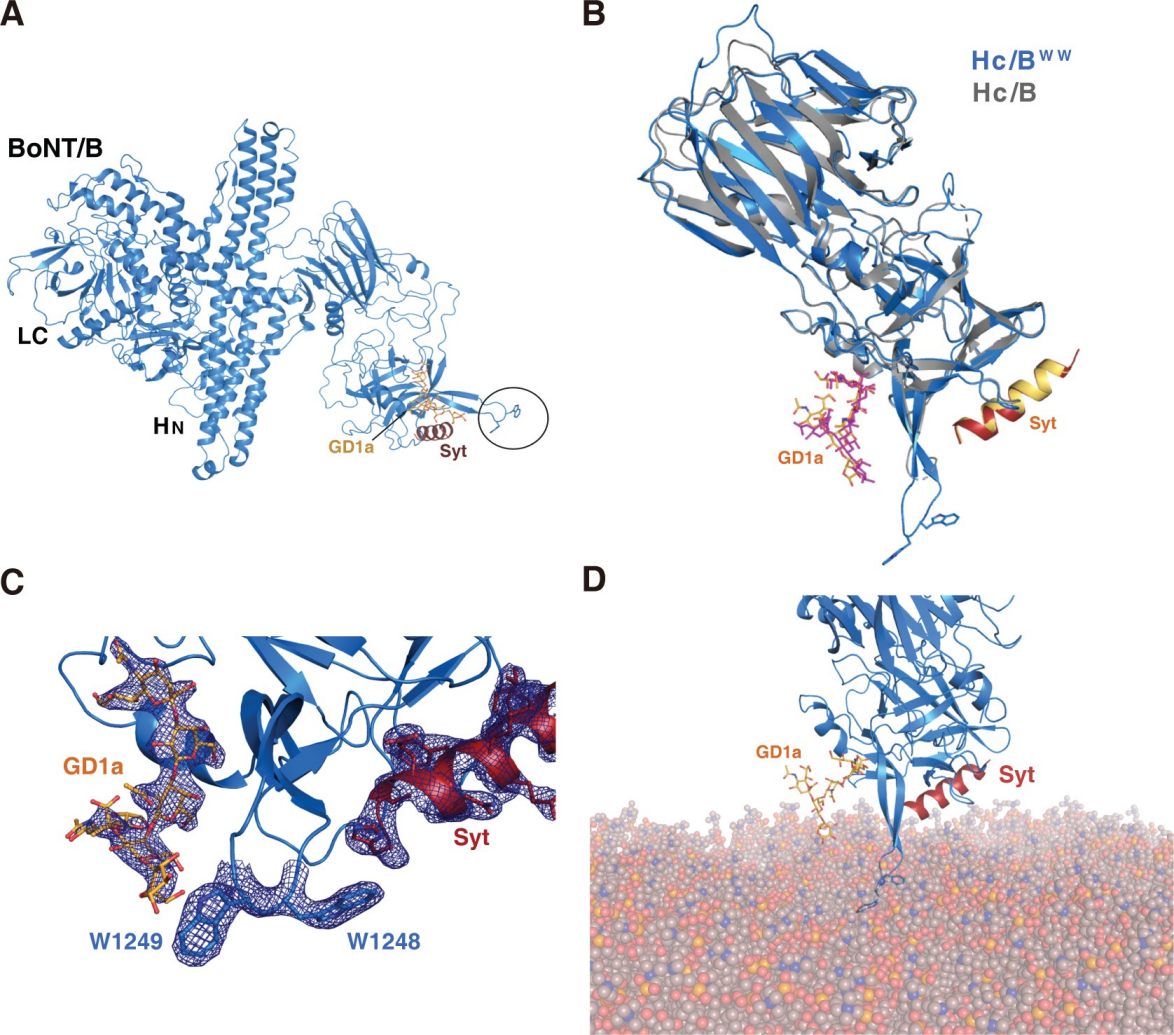
Cocrystal structure of H_C_/B^WW^ in complex with Syt I peptide and ganglioside oligosaccharide. (A) Cocrystal structure of H_C_/B^WW^ (blue) in complex with hSyt I peptide (purple) and ganglioside (yellow). The LC and H_N_ were added from superposition with the full-length BoNT/B (PDB 1EPW). Location of the WW mutations is circled. (B) Overlay of the crystal structures of WT H_C_/B (gray, PDB 4KBB) and H_C_/B^WW^ (blue), with the ganglioside oligosaccharide (pink and yellow, from the respective structures) and hSyt I (red)/rSyt II (yellow). The two mutated W residues are shown as sticks. (C) The region containing the GBS, Syt-binding site, and LBL is enlarged, with electron density (F_o_-F_c_ map at 1σ) of GD1a, Syt I, and the two mutated W residues shown as a blue mesh. (D) H_C_/B^WW^ is modeled onto membranes through anchoring with both GD1a and Syt I, showing that the two W residues in its LBL interact with membranes. BoNT, botulinum neurotoxin; GBS, ganglioside-binding site; GD1a, disialoganglioside; H_C_, C-terminal receptor-binding domain; H_N_, N-terminal translocation domain; hSyt I, human Syt I; LBL, lipid-binding loop; LC, light chain; PDB, Protein Data Bank; rSyt II, rat Syt II; Syt, synaptotagmin; WT, wild-type.

Finally, to further exclude the possibility that WW mutations may have unexpected effects on the structure/function of H_C_/B, we sought to solve the cocrystal structure of H_C_/B^WW^ in complex with both gangliosides and Syt. Crystals were successfully obtained for H_C_/B^WW^ in the presence of the GD1a oligosaccharide and a peptide corresponding to human Syt I (residues 33–53, hSyt I). The structure was solved to a 2.4-Å resolution (S2 Table). The overall structure of H_C_/B^WW^ is virtually identical to that of WT H_C_/B (Fig 6A). The interactions of H_C_/B^WW^ with GD1a and hSyt I also match the previously solved structure of H_C_/B in complex with GD1a and rat Syt II peptides (Fig 6B) [25]. Recognition of GD1a is based on a stacking interaction between W1262 and the central galactose-4 moiety of the carbohydrate, with the surrounding pocket (including E1190, H1241, S1260, Y1263, and E1273) offering multiple hydrogen bonds to the adjacent N-acetylglucosamine (GalNAc3) and sialic acid group (Sia5) (S6A Fig). On the other side of the LBL, the Syt peptide occupies the binding crevice and mostly involves hydrophobic interactions. Superposition of the H_C_/B^WW^:hSyt I and H_C_/B:rSyt II structures clearly shows that the WW mutations did not alter the protein receptor binding site (Fig 6B and S6B Fig).

The LBL was clearly defined in the H_C_/B^WW^ structure and is located between the GBS and Syt-binding site (Fig 6B and 6C). The loop protrudes significantly, with the side chains of W1248 and W1249 exposed toward the surface at the tip of LBL in H_C_/B^WW^. The hydrophobic loop of H_C_/B^WW^ is located in a similar position to the one in H_C_/DC, where residues 1251–1253 (YWF) protrude into the membrane bilayer (S6C Fig). These results demonstrate that introducing two tryptophan residues at this position does not alter the structure of H_C_/B and only extends the LBL with two surface-exposed aromatic residues. Using the extracellular domains of GD1a and hSyt I as two anchoring points, we manually docked H_C_/B^WW^ onto the surface of lipid membranes, which illustrated how the two tryptophan residues would penetrate into lipid membranes, thus providing a structural basis for H_C_/B^WW^ binding to lipid membranes (Fig 6D).

## Discussion

The role of LBLs in BoNT/B, C, D, G, and DC for binding to lipid membranes has been hypothesized previously. Mutagenesis studies have demonstrated the importance of LBL for forming stable binding to membrane-embedded receptors and for the potency of these toxins [64–68, 71]. However, it has been challenging to detect direct binding of these toxins to lipid membranes because protein−lipid interactions are usually transient and of rather low binding affinity. By combining a nanodisc model with BLI assays, we were able to detect direct binding of H_C_/DC, H_C_/C, and H_C_/G, as well as H_C_/B^W^ and H_C_/B^WW^, to lipid membranes. In contrast, H_C_/B and H_C_/D showed no detectable lipid-binding capability under our assay conditions. To provide a stable baseline for detecting weak H_C_–lipid interactions, we utilized high concentrations of H_C_s (e.g., 50 μM) and biotin-labeled lipids to immobilize nanodiscs to the biosensors.

Aromatic residues such as tyrosine, tryptophan, and phenylalanine have the capability to insert their bulky aromatic rings into the hydrophobic lipid bilayer while keeping their hydrophilic side chain groups in the aqueous environment [75]. Therefore, they are ideal choices for mediating interactions with lipid membranes at the aqueous–lipid interface. Tryptophan has the strongest effect, whereas phenylalanine is the weakest of the three aromatic residues [75]. H_C_/DC contains three (YWF) and H_C_/C contains two (WY) aromatic residues at the tip of their LBLs, which appears to be the optimal design for mediating lipid interactions. The number and composition of aromatic residues both influence overall binding affinity. For instance, mutating any one of the three aromatic residues in H_C_/DC reduced its binding to lipids, whereas changing the F residue to W increased binding. The aromatic residues are separated in H_C_/D (WRFSF) and H_C_/G (YGYVW), which might be the reason for their loss of or low lipid-binding affinity. H_C_/B-LBL contains only a single aromatic residue (F), which is also away from the tip of LBL; thus, H_C_/B showed no detectable lipid-binding capability.

The ternary complex of H_C_/B^WW^ with both gangliosides and Syt I peptide demonstrates conclusively that introducing two consecutive tryptophan residues does not alter the conformation of H_C_/B or affect the GBS and Syt-binding site. The only change is the extension of H_C_/B-LBL with two tryptophan residues at the tip. These data provided strong support for the role of consecutive aromatic residues at the tip of LBL in mediating lipid binding. The ternary structure also confirms that LBL is separate from the GBS and the Syt-binding site, suggesting that it contributes to overall binding affinity toward neuronal membranes independently.

Examining binding of H_C_/B^WW^ to nanodiscs with different receptor components suggests that LBL–lipid interactions are more relevant to synergizing with ganglioside binding, with minimal impact on Syt binding for H_C_/B. This is likely because the H_C_/B-Syt interactions already have high affinity (approximately 10^−7^ M range), whereas H_C_/B–ganglioside interactions have low affinity (approximately 10^−5^ M range), making the impact of adding LBL–lipid interactions more obvious for ganglioside binding. Because binding of H_C_/DC to Syt II has a lower affinity (approximately 10^−5^ M range) than H_C_/B-Syt interactions, the synergistic effect with LBL–lipid interactions is obvious for H_C_/DC binding to membrane-embedded Syt II: WT H_C_/DC showed approximately 10-fold higher binding affinity toward ND-Syt compared with H_C_/DC containing mutations at the tip aromatic residues (Y1251A, W1252A, or F1253A). These point mutations in H_C_/DC showed the same level of binding affinity toward ND-Syt as with isolated Syt II, indicating that mutations in LBL do not affect H_C_/DC binding to membrane-embedded Syt II. Thus, their lower binding affinity toward ND-Syt compared with WT H_C_/DC is due to loss of LBL–lipid interactions. It is interesting to note that H_C_/DC has the highest level of LBL–lipid interactions, which may compensate its lower binding affinity toward Syt I/II compared with H_C_/B.

H_C_/B^WW^ showed enhanced binding to neuronal surfaces than WT H_C_/B. The binding remains specific to nerve terminals rather than being diffused along neuronal membranes, and binding of H_C_/B^WW^ to neurons was largely abolished by depleting CGs and by competition with a soluble Syt II fragment containing the toxin-binding site. This is likely because LBL–lipid interactions and H_C_/B–ganglioside interactions are both relatively weak, which allows BoNT/B to sample cell surfaces through fast-on/fast-off transient interactions. Only when Syt I/II are also present can the toxin be stably anchored to nerve terminals.

Finally, in vivo DAS assays revealed two major advantages of BoNT/B^MY-WW^ over the control BoNT/B^MY^: (1) The safety ratio was improved: this is likely because BoNT/B^MY-WW^ has less unwanted diffusion from the injection site. (2) There was longer therapeutic duration compared with the same dose of BoNT/B^MY^: this is likely because more BoNT/B^MY-WW^ can enter the targeted local neurons than BoNT/B^MY^, which is also consistent with the suggestion that BoNT/B^MY-WW^ has less diffusion and may thus remain near the injection site longer than BoNT/B^MY^. BoNT/B is currently utilized in the clinic as a therapeutic toxin and has the advantage of targeting smooth muscles more efficiently than the more widely used BoNT/A. Introducing lipid-binding capability to BoNT/B and potentially other BoNTs may present new ways to engineer toxins with improved therapeutic efficacy and reduced side effects.

## Materials and methods

### Ethics statement

All animal studies were approved by the Boston Children’s Hospital Institutional Animal Care and Use Committee (protocol number: 18-10-3794R). The Boston Children’s Hospital institutional animal care program holds an active animal welfare assurance on file with the Office of Laboratory Animal Welfare at National Institutes of Health, which describes Children’s adherence to Public Health Service’s Policy on Humane Care and Use of Laboratory Animals. It is also registered as a research facility with the US Department of Agriculture and follows the Animal Welfare Act and Regulations, when applicable. In addition, Children’s animal care and use program is fully accredited by AAALAC International.

### Biosafety

All procedures were approved by the Institute of Biosafety Committees at Boston Children’s Hospital (protocol number: IBC-P00000501). No part of the study has been deemed classified information. All active BoNTs are stored in a locked freezer. Used toxins and contaminated media/reagents/containers are exposed to 10% bleach solution for decontamination.

### Constructs and protein purifications

cDNAs encoding H_C_/B, H_C_/C, H_C_/D, H_C_/DC, and H_C_/G were subcloned into pET28a vector with an N-terminal HA tag. A modified version of ApoA1 was cloned into pNFXeX vector and expressed as an N-terminal His6-tagged protein [78, 79]. cDNA encoding mouse Syt II 1–267 was subcloned into the pET28a vector and expressed as an N-terminal His6-tagged protein. cDNA encoding BoNT/B^MY^ was subcloned into pET32a as previously described [62]. A stop codon was introduced to remove the C-terminal His6-tag. cDNA encoding mouse Syt II 1–61 was subcloned into the pGEX-4T-1 vector and expressed as N-terminal GST-tagged fusion proteins. All mutated proteins were generated using QuikChange site-directed mutagenesis. All proteins were expressed in *E*. *coli* BL21(DE3) strains. H_C_ protein expression was induced using IPTG at 22°C for 16 hours. The full-length BoNT/B (BoNT/B^MY^ and BoNT/B^MY-WW^) mutants were induced using IPTG at 16°C for 20 hours. For all other proteins, expression was induced using IPTG at 18°C for 16 hours. Cells were harvested at 4°C and lysed by sonication. Cell lysates were centrifuged for 60 minutes at 4°C and then filtered using syringe-driven filters (Argos Technologies, 0.22 μm). After centrifugation and filtration, harvested supernatants were loaded into Nickel-NTA columns (GE) using the AKTA Prime FPLC system (GE). Proteins were eluted using imidazole gradient elution and further desalted using the PD-10 column. For mouse Syt II 1–267, the cell lysis buffer contains 1% (v/v) Triton X-100, and the protein elution buffer contains 0.8% (W/V) n-octyl β-D-glucopyranoside (OG). For GST-tagged mSyt II 1–61, harvested supernatant was incubated with GST beads at 4°C for 1 hour. The beads were washed with phosphate-buffered saline (PBS), and then the protein was eluted with PBS containing 10 mM reduced L-Glutathione (GSH reduced). All the proteins used in the BLI assay were desalted in PBS.

For full-length BoNT/B mutants (BoNT/B^MY^ and BoNT/B^MY-WW^), cells were harvested at 4°C and lysed by sonication in 50 mM Tris (pH 8), 200 mM NaCl, and treated with benzonase. Cell lysates were adjusted to 1.3 M (NH_4_)_2_SO_4_ and centrifuged for 60 minutes at 4,000*g*. The recovered supernatants were loaded onto a Butyl HP (GE) column using an AKTA Pure system (GE). Proteins were eluted with a 1.3–0 M (NH_4_)_2_SO_4_ in 50 mM Tris (pH 8) linear gradient. Fractions containing the target protein were pooled and desalted using a HiPrep Sephadex G-25 desalting column (GE). Further purification was performed by anion exchange chromatography with a Q HP HiTrap column (GE). Proteins were eluted with a 0–1 M NaCl in 50 mM Tris (pH 8) linear gradient. The fractions containing target protein were pooled and stored at −80°C.

### Nanodisc preparation and purification

All lipids were purchased from Avanti Polar Lipids (Alabaster, AL). For ND1 and ND-Syt, the lipid mixture composition used was POPC/DOPS/cholesterol/biotin-PEG-DSPE (molar ratio: 74.5:5:20:0.5). For ND-CGs and ND-Syt-CGs, the lipid mixture composition used was POPC/DOPS/cholesterol/biotin-PEG-DSPE/GD1a/GT1b (molar ratio: 72.5:5:20:0.5:1:1). For nanodisc without biotin, the lipid mixture composition used was POPC/DOPS/cholesterol/ GD1a/GT1b (molar ratio: 73:5:20:1:1). For POPC ND, the lipid mixture composition used was POPC/biotin-PEG-DSPE (molar ratio: 99.5:0.5).

The lipids dissolved in chloroform solution were first diluted and subsequently mixed together in glass tubes under a fume hood according to the aforementioned molar ratio. Then, the solvent in the mixtures was dried under nitrogen gas in a fume hood, and the remaining solvent was further removed at room temperature (22–24°C) overnight in a vacuum desiccator. The lipid films were resuspended in PBS with agitation. The final lipid stock concentration was 50 mM.

For ND1 and ND-CGs, sodium cholate was used to solubilize lipids and then mixed with ApoA1 at a molar ratio of 60:1. For ND-Syt and ND-Syt-CGs, sodium cholate–solubilized lipids were mixed with ApoA1 and mouse Syt II 1–267 at a molar ratio of 60:1:1. Formation of nanodiscs was then triggered by mixing with methanol-activated SM2 Bio-Beads for 4 hours, followed by another round of mixing with new SM2 Bio-Beads for another 4 hours. Then, the nanodiscs were filtered using Costar Spin-X Plastic centrifuge tube filters (0.22-μm membrane pore size) and further purified using FPLC (Bio-Rad NGC chromatography systems) using a Superdex 200 GL 10/300 column (GE Healthcare). The formation of nanodiscs was confirmed and purity assessed using SDS-PAGE gel analysis and negative staining EM.

### Negative staining EM

EM grids (Formvar Carbon Support Film on Square Grids, Electron Microscopy Sciences: square grid, material = Copper, thickness = 10/1 nm, mesh = 400) were first pretreated in a glow discharge unit for 2 minutes to allow adsorption of samples, after which 2 μl of the nanodisc samples was added to the grid surface. After 30 seconds, the sample droplets were adsorbed using filter paper on the edge of the grid. Then, the grids were soaked into 1.5% uranyl formate solution droplet for 30 seconds and dried using filter paper. The grids were further cleaned using 1.5% uranyl formate solution three times. Air-dried grids were then imaged on the Tecnai G2 Spirit BioTWIN transmission electron microscope and recorded with an AMT 2k CCD camera at the Harvard Medical School Electron Microscopy Facility.

### Biolayer interferometry BLItz system assay

For SA biosensor experiments, the Pall ForteBio's SA biosensors were prehydrated for 10 minutes. The nanodiscs containing biotin-lipids were loaded onto the biosensors, which were equilibrated in PBS for 400 seconds, and then exposed to solutions containing H_C_ for 40 seconds as the association step. The biosensors were then transferred to PBS for an 80-second dissociation step.

For anti-GST biosensor experiments, the Pall ForteBio's anti-GST biosensors were prehydrated for 10 minutes. M-Syt II 1–61 protein with a GST tag was first loaded onto the anti-GST biosensor, followed by equilibration in PBS for 400 seconds and then exposure to proteins for 40 seconds as the association step. The biosensors were then transferred to PBS for 80 seconds as the dissociation step.

For both experiments, 0.5% BSA was added to all buffers to reduce nonspecific binding. Data were analyzed using the data analysis software BLItz Pro Software v8.1 (Pall ForteBio). To calculate the total binding response during the association step, the last five signals (the last 500 milliseconds) of the association phase were averaged as the final response signal. The baseline value was subtracted from the final response signal to generate the 40-second response. The 40-second response curve was plotted using the GraphPad Prism software. In the response curve, the concentration was plotted along the x-axis, and the response was plotted along the y-axis. K_D_ values were calculated using the data analysis software BLItz Pro Software v8.1 following the BLItz system user guide (Pall ForteBio).

### Biolayer interferometry Octet RED 384 system assay

For Biolayer Interferometry Octet RED 384 system assay, sample solutions and buffers were prepared in black 384-well plates. The program used was precompiled and run automatically during the experiments as follows: The Pall ForteBio's SA biosensors were prehydrated for 10 minutes. The nanodiscs with a biotin tag were loaded onto the biosensors for 400 seconds, equilibrated in PBS for another 500 seconds, and then exposed to H_C_ solutions for 800 seconds as the association step, followed by incubation in PBS for 800 seconds as the dissociation step. The experiments were carried out at 37°C with 1,000-rpm shaking. During all the experiments, all buffers contained 0.5% BSA. The data were collected using the data acquisition software (Pall ForteBio) and analyzed using the data analysis HT11.0 software (Pall ForteBio). During the data analysis, the data were aligned to the baseline step, and high-frequency noise was removed with Savitzky-Golay filtering. The 1:1 binding model was used for fitting. K_D_ values were calculated using the data analysis HT11.0 software (Pall ForteBio).

### Liposome flotation assay

POPC and gangliosides were purchased from Avanti Polar Lipids (Alabaster, AL). POPC or POPC mixed with 1% ganglioside mixture was dried with nitrogen gas and further dried overnight at room temperature under vacuum to form the lipid films. The lipids were then resuspended with Tris buffer. The resuspended lipids were extruded through a polycarbonate filter (Whatman, 200-nm pore size) by 20 strokes to form liposomes. Liposomes (75 μl) were incubated with H_C_/B proteins (1 μM) at room temperature for 30 minutes. The liposome–protein mixture was then added to sucrose solution to reach a final sucrose concentration of 30%, which served as the bottom layer in the centrifuge tube. The 200 μl of 25% sucrose was loaded on the top of the bottom layer, followed by 50 μl of Tris buffer. Loaded samples with sucrose gradient were centrifuged at 240,000*g* for 1 hour (Beckman TLS-55 rotor, OptiMax MAX-XP benchtop centrifuge). After centrifugation, 50-μl solutions on the top of the gradient were collected and subjected to immunoblot analysis.

### Neuron culture

Mouse cortical neurons were prepared from newborn pups (Consortium for Functional Glycomics). Rat cortical neurons were prepared from E18–19 embryos dissected from pregnant rats (Sprague Dawley strain, purchased from Charles River). Dissected cortices were dissociated with papain according to the manufacturer’s instructions (Worthington Biochemical). Cells were plated on poly-D-lysine-coated coverslips in 24-well plates. Experiments were carried out using days in vitro (DIV) 11–13 for mouse neurons and DIV 13–15 for rat neurons.

### Immunoblot analysis and Immunofluorescence staining

Neurons were exposed to 100 nM H_C_/B in high-K^+^ buffer containing 87 mM NaCl, 56 mM KCl, 1.5 mM KH_2_PO_4_, 8 mM Na_2_HPO_4_, 0.5 mM MgCl_2_, and 1 mM CaCl_2_ for 5 minutes at 37°C. Cells were then washed three times with PBS. Binding of H_C_/B was examined with two complementary approaches: (1) For immunostaining, neurons were fixed with 4% paraformaldehyde and permeabilized with 0.3% Triton X-100 in PBS. Images were collected using a Zeiss 880 laser scanning confocal microscope with a 40× oil objective. At least three representative images were collected per condition. (2) For immunoblot analysis, the final concentration of proteins for neuron binding is 0.1 μM, and the total volume is 200 μl. Neurons in each well were lysed in a lysis buffer (PBS with 1% Triton X-100, 0.05% SDS and protease inhibitor cocktail [Roche], 80 μL per well in 24-well plates). The 20-μl 5× loading buffer was mixed with the supernatant of lysed cells. Samples were heated to 56°C for 5 minutes, lysates were centrifuged for 10 minutes at 4°C, and then 5-μl supernatants were subjected to SDS-PAGE and western blot analysis. Binding of H_C_/B was detected using a monoclonal human anti-HA antibody. The antibodies used were purchased from the indicated vendors: mouse monoclonal antibody for actin (Clone AC-15, Sigma), mouse monoclonal anti-HA (16B12, Covance), and rabbit polyclonal antisynapsin (Millipore).

### DAS assay

The control BoNT/B^MY^ and BoNT/B^MY-WW^ toxins both contain two point mutations E1191M/S1199Y [61]. They were activated by trypsin digestion and then reconstituted in phosphate buffer containing 0.2% gelatin (pH 6.3), and serial dilutions were prepared based on preliminary dose-response analysis. The following final concentrations were tested in DAS assay: 8, 10, 12.5, 19.7, and 24.7 pg/mouse. The following final concentrations were tested in IM LD50 assay: 27.7, 29.0, 30.5, 32, 33.7, 35, 37, and 39 pg/mouse. The 5-μl diluted toxins were injected into the right gastrocnemius muscle of each mouse.

### H_C_/B^WW^ crystallization and structure determination

The H_C_/B^WW^ construct used for crystallization was subcloned into a pET28a vector and expressed as an N-terminal His6-tagged protein. Cultures were grown at 37°C, and induction was carried out at 18°C using 0.5 mM IPTG. Bacterial pellets were lysed by sonication, and the protein was purified by affinity chromatography using a 5-ml HisTrap HP column (GE Healthcare) followed by gel filtration with a 16/60 Superdex 200 column (GE Healthcare). The final sample was stored at 11.3 mg/ml in 20 mM HEPES (pH 7.5), 300 mM NaCl, 10% glycerol, and 2 mM TCEP. A sample consisting of H_C_/B^WW^ (10 mg/ml) mixed with 5 mM GD1a oligosaccharide (Elicityl, France) and 1 mM of the peptide corresponding to human Syt I (33–53) was prepared. Crystals of the complex grew within 3 days at 21°C using the sitting drop vapor-diffusion method, in which 200 nl of sample was mixed with 100 nl of reservoir solution composed of 20% v/v PEG6000, 0.1 M MES (pH 6.0), and 0.2 M sodium chloride. The crystals were transferred into a cryoprotectant solution supplemented with 15% v/v glycerol and then frozen in liquid nitrogen for data collection. Diffraction data were collected at station I04 of the Diamond Light Source (Didcot, United Kingdom) equipped with a PILATUS-6M detector (Dectris, Switzerland). A complete data set to 2.4 Å was collected from a single crystal at 100 K. Raw data images were processed and scaled with DIALS [80] and AIMLESS [81] using the CCP4 suite 7.0 [82]. The structure was solved using molecular replacement with the coordinates of the H_C_/B dual receptor complex (PDB code 4BKZ) as a search model in PHASER [83]. The working models were refined using REFMAC5 [84] and manually adjusted with COOT [85]. Water molecules were added with the help of ARP/wARP [86] at positions where Fo-Fc electron density peaks exceeded 3σ and potential hydrogen bonds could be made. Validation was performed with MOLPROBITY [87]. Crystallographic data statistics are summarized in S2 Table. All figures were drawn with PyMOL (Schrödinger, NY). The atomic coordinates and structure factors (code 6QNS) have been deposited in the Protein Data Bank (http://wwpdb.org).

## Supporting information

S1 Fig Nanodisc preparation, purification, and characterization. (A) Representative FPLC elution profiles of nanodiscs (Superdex 200GL 10/300 column) and SDS-PAGE gel/Coomassie Blue staining analysis validated the presence of ApoA1 proteins in the collected nanodisc fraction. Lane 1 on the left represents the protein ladder. (B) Representative FPLC elution profiles of nanodiscs containing Syt II and SDS-PAGE gel/Coomassie Blue staining analysis validated the incorporation of Syt II in the collected nanodisc fraction. (C) Schematic drawing of nanodiscs (ND-CGs, ND-Syt, and ND-Syt-CGs) and negative staining EM showing the presence of nanodiscs. Scale bar represents 25 nm. (D) Nanodiscs were loaded to SA biosensors for BLI. All nanodiscs showed robust binding (association) with virtually no dissociation. (E) The presence of BSA (0.5%) further reduces background binding of nanodiscs lacking biotin-DSPE to SA biosensors. Top panel: ND-CGs without biotin-DSPE; bottom panel: ND-Syt-CGs without biotin-DSPE. ApoA1, Apolipoprotein A1; BLI, biolayer interferometry; BSA, bovine serum albumin; CG, complex ganglioside; DSPE, 1,2-distearoyl-sn-glycero-3-phosphoethanolamine; EM, electron microscopy; FPLC, fast protein liquid size-exclusion chromatography; ND, nanodisc; SA, streptavidin; SDS-PAGE, sodium dodecyl sulfate–polyacrylamide; Syt, synaptotagmin.
(TIF)

S2 Fig Binding of H_C_/B, H_C_/C, H_C_/D, H_C_/DC, and H_C_/G to POPC nanodiscs. (A) Schematic drawing of nanodiscs containing only POPC (POPC ND). (B–F) POPC ND was immobilized to biosensors. Binding of H_C_/B (panel B), H_C_/C (panel C), H_C_/D (panel D), H_C_/DC (panel E), and H_C_/G (panel F) to POPC ND was measured by BLI assays. H_C_/B and H_C_/D showed no detectable binding to POPC ND. H_C_/DC showed the strongest binding, and H_C_/C showed modest levels, whereas H_C_/G showed low levels of binding to POPC ND. BLI, biolayer interferometry; H_C_, C-terminal receptor-binding domain; ND, nanodisc; POPC, 1-palmitoyl-2-oleoyl-glycero-3-phosphocholine.
(TIF)

S3 Fig Binding of H_C_/DC to ND-Syt and free Syt II. (A) Binding of WT H_C_/DC and the indicated H_C_/DC mutants to ND-Syt was analyzed by BLI assays. Binding kinetics were analyzed and are listed in Table 1. (B) Binding of WT H_C_/DC and the indicated H_C_/DC mutants to immobilized GST-tagged Syt II (1–61) protein was analyzed by BLI assays. The binding kinetics were analyzed and listed in Table 1. Mutating the tip residues in H_C_/DC-LBL does not alter its binding to Syt II. BLI, biolayer interferometry; GST, glutathione S-transferase; H_C_, C-terminal receptor-binding domain; LBL, lipid-binding loop; ND, nanodisc; Syt, synaptotagmin; WT, wild-type.
(TIF)

S4 Fig Engrafting the LBL sequence of H_C_/DC onto H_C_/B did not result in binding to membranes. (A) Superposition of the LBLs of H_C_/DC and H_C_/B. (B) Sequence changes in H_C_/B. (C) The indicated H_C_/B mutants were examined for binding to ND1 using BLI at 10 μM concentrations. BLI, biolayer interferometry; H_C_, C-terminal receptor-binding domain; LBL, lipid-binding loop; ND1, receptor-free nanodisc.
(TIF)

S5 Fig Adding three aromatic residues to H_C_/B-LBL further increases its binding to membranes but reduces solubility. (A) Binding of the indicated mutants to ND1 was analyzed using BLI at 10 μM concentration. The specific mutations in LBL are marked in red. (B) The maximal binding signals of H_C_/B mutants (10 μM) to ND1 were plotted in the bar graph. Error bars indicate means ± SD, *n* = 3. Numerical values for (B) are available in S1 Data. BLI, biolayer interferometry; H_C_, C-terminal receptor-binding domain; LBL, lipid-binding loop; ND1, receptor-free nanodisc.
(TIF)

S6 Fig Cocrystal structure of H_C_/B^WW^ in complex with Syt I peptide and ganglioside oligosaccharide. (A) The ganglioside-binding site. GD1a in the WT costructure in pink, and blue in H_C_/B^WW^ complex. Main residues involved in binding are shown as sticks, with hydrogen bonds represented as dashed lines. (B) The Syt-binding site. Residues involved in binding are highlighted, illustrating the high similarity between the mutant and WT H_C_/B bound to hSyt I (red) and rSyt II (yellow), respectively. (C) The structures of H_C_/DC (cyan) in complex with hSytI (PDB 4isq) and the sialyl-T carbohydrate (PDB 5lr0) shows that the LBL loop (residues 1251–1253) interacts with the membrane. GD1a, disialoganglioside; H_C_, C-terminal receptor-binding domain; hSyt I, human Syt I; LBL, lipid-binding loop; PDB, Protein Data Bank; rSyt II, rat Syt II; Syt, synaptotagmin; WT, wild-type.
(TIF)

S1 Table Kinetics and affinity analysis of WT H_C_/B and H_C_/B^WW^ binding to nanodiscs. H_C_, C-terminal receptor-binding domain; WT, wild-type.
(DOCX)

S2 Table X-ray crystallography: Data collection and refinement statistics. (DOCX)

S1 Data Data underlying Figs 1I, 2E, 3D, 5A, 5D and S5B. (XLSX)

S1 Raw Images Raw gel images of western blotting or Coomassie blue staining data included in Figs 3F, 5A, S1A and S1B. (TIF)

## Abbreviations

ApoA1: Apolipoprotein A1
BLI: biolayer interferometry
BoNT: botulinum neurotoxin
BSA: bovine serum albumin
BW: body weight change
CG: complex ganglioside
DAS: Digit Abduction Score
DOPS: 1,2-dioleoyl-sn-glycero-3-phospho-L-serine
DSPE: 1,2-distearoyl-sn-glycero-3-phosphoethanolamine
ED50: median effective dose
EM: electron microscopy
FPLC: fast protein liquid size-exclusion chromatography
GalNAc: N-acetyl galactosamine
GBS: ganglioside-binding site
GD1a: disialoganglioside
GD2 KO: *Galgt1* knockout
GST: glutathione S-transferase
GT1b: trisialoganglioside
HA: human influenza hemagglutinin
H_C_: C-terminal receptor-binding domain
HC: heavy chain
H_N_: N-terminal translocation domain
hSyt I: human Syt
IM LD50: intramuscular median lethal dose
ka: association rate constant
kd: dissociation rate constant
K_D_: equilibrium dissociation constant
LBL: lipid-binding loop
LC: light chain
MPN: mouse phrenic nerve hemidiaphragm assay
MSP: membrane scaffolding protein
ND: nanodisc
ND1: receptor-free nanodisc
NSF: N-ethylmaleimide-sensitive factor
PC: phosphatidylcholine
PDB: Protein Data Bank
POPC: 1-palmitoyl-2-oleoyl-glycero-3-phosphocholine
PMP1: Paraclostridial Mosquitocidal Protein 1
rSyt II: rat Syt II
SA: streptavidin
Sia: sialic acid group
SNAP-25: synaptosomal nerve-associated protein 25
SNARE: soluble NSF attachment protein receptor
SV2: synaptic vesicle protein 2
Syt: synaptotagmin
VAMP: vesicle associated membrane protein
WT: wild-type
