# Supplementary figures and images for: Characterization of a membrane binding loop leads to engineering botulinum neurotoxin B with improved therapeutic efficacy
Source: PLoS Biol. 2020 Mar 17;18(3):e3000618. doi: 10.1371/journal.pbio.3000618 (PMC7077807; doi:10.1371/journal.pbio.3000618)

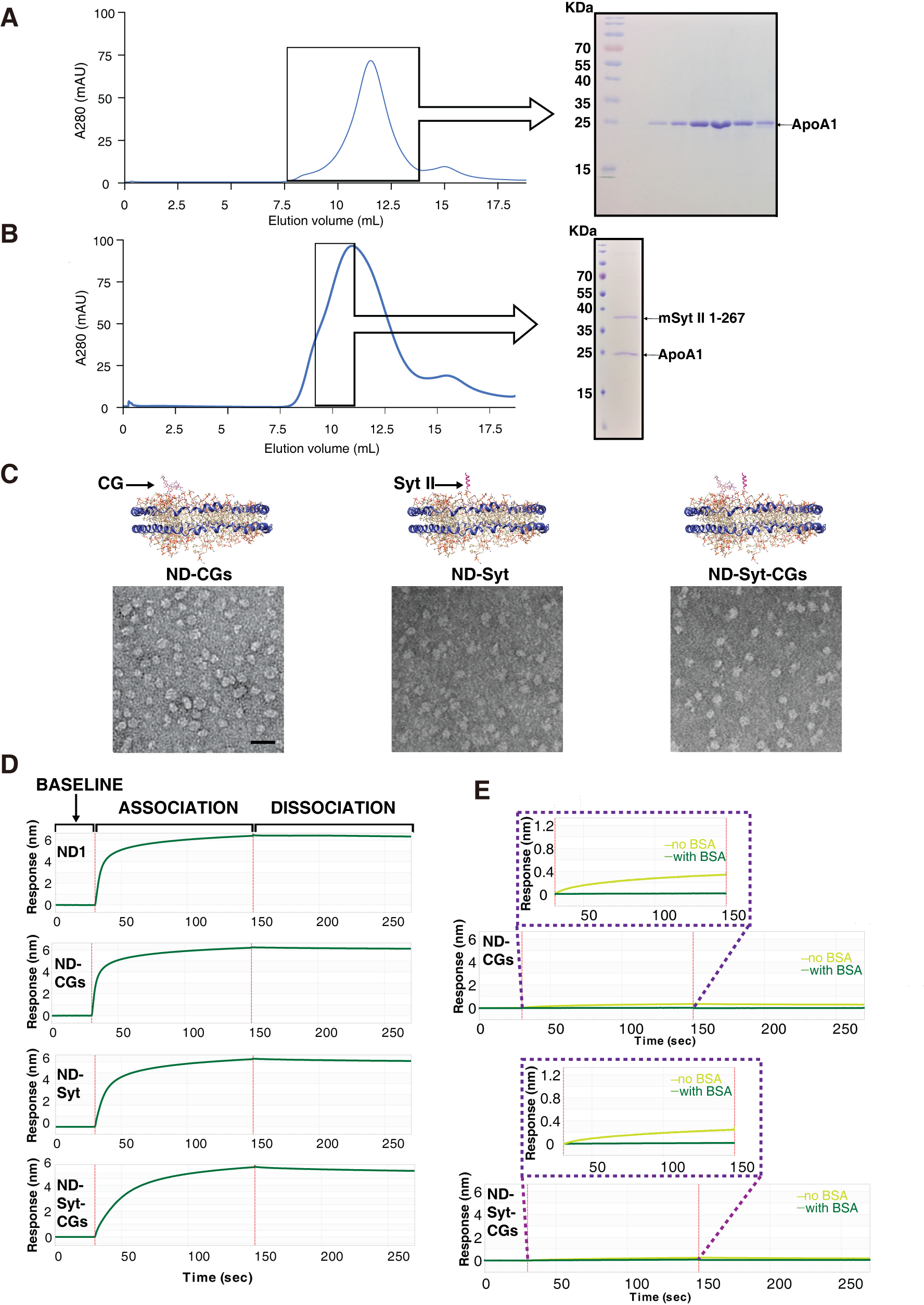

Supplement: S1 Fig — (A) Representative FPLC elution profiles of nanodiscs (Superdex 200GL 10/300 column) and SDS-PAGE gel/Coomassie Blue staining analysis validated the presence of ApoA1 proteins in the collected nanodisc fraction. Lane 1 on the left represents the protein ladder. (B) Representative FPLC elution profiles of nanodiscs containing Syt II and SDS-PAGE gel/Coomassie Blue staining analysis validated the incorporation of Syt II in the collected nanodisc fraction. (C) Schematic drawing of nanodiscs (ND-CGs, ND-Syt, and ND-Syt-CGs) and negative staining EM showing the presence of nanodiscs. Scale bar represents 25 nm. (D) Nanodiscs were loaded to SA biosensors for BLI. All nanodiscs showed robust binding (association) with virtually no dissociation. (E) The presence of BSA (0.5%) further reduces background binding of nanodiscs lacking biotin-DSPE to SA biosensors. Top panel: ND-CGs without biotin-DSPE; bottom panel: ND-Syt-CGs without biotin-DSPE. ApoA1, Apolipoprotein A1; BLI, biolayer interferometry; BSA, bovine serum albumin; CG, complex ganglioside; DSPE, 1,2-distearoyl-sn-glycero-3-phosphoethanolamine; EM, electron microscopy; FPLC, fast protein liquid size-exclusion chromatography; ND, nanodisc; SA, streptavidin; SDS-PAGE, sodium dodecyl sulfate–polyacrylamide; Syt, synaptotagmin. (TIF) [file pbio.3000618.s001.tif]

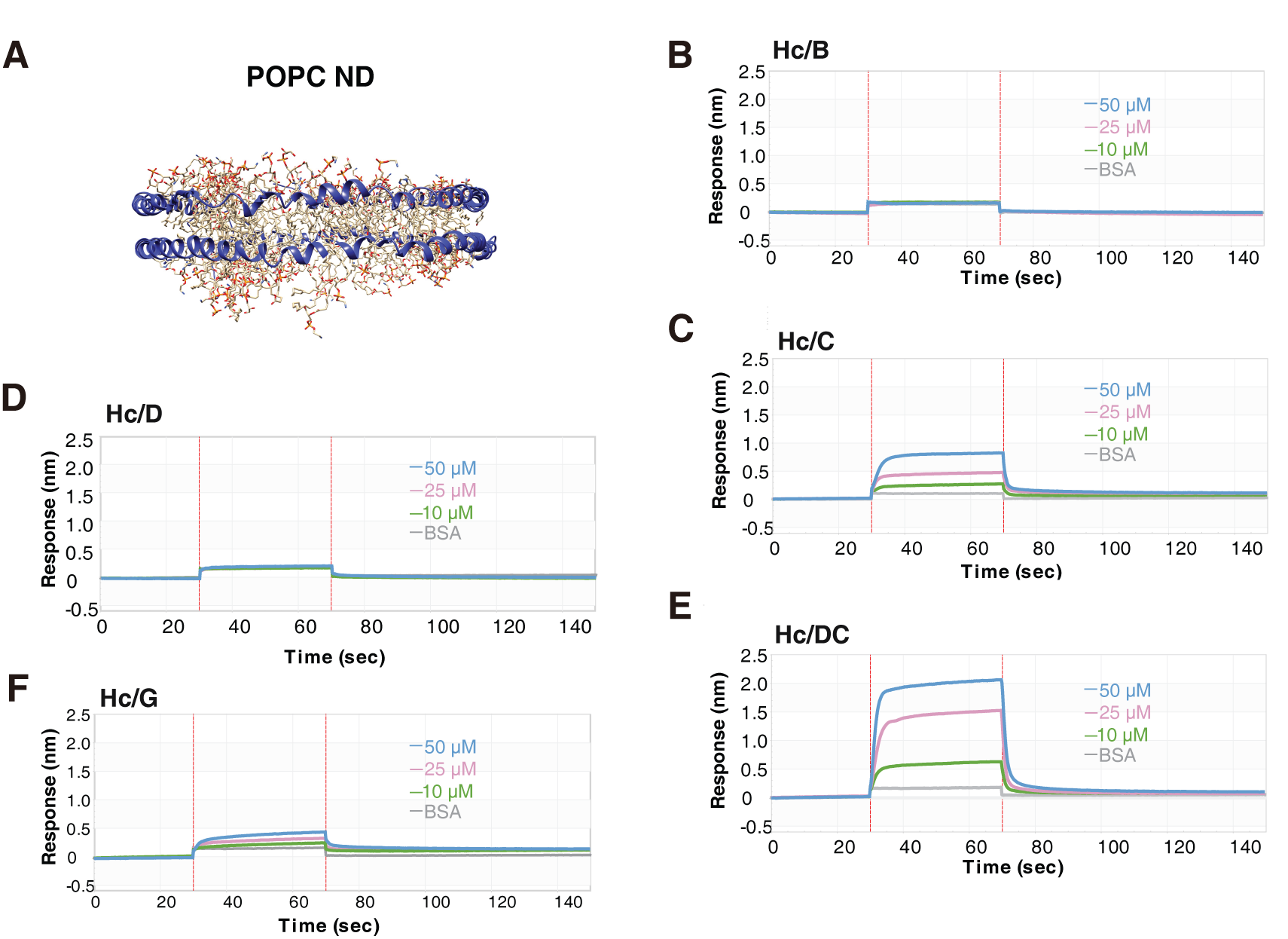

Supplement: S2 Fig — (A) Schematic drawing of nanodiscs containing only POPC (POPC ND). (B–F) POPC ND was immobilized to biosensors. Binding of HC/B (panel B), HC/C (panel C), HC/D (panel D), HC/DC (panel E), and HC/G (panel F) to POPC ND was measured by BLI assays. HC/B and HC/D showed no detectable binding to POPC ND. HC/DC showed the strongest binding, and HC/C showed modest levels, whereas HC/G showed low levels of binding to POPC ND. BLI, biolayer interferometry; HC, C-terminal receptor-binding domain; ND, nanodisc; POPC, 1-palmitoyl-2-oleoyl-glycero-3-phosphocholine. (TIF) [file pbio.3000618.s002.tif]

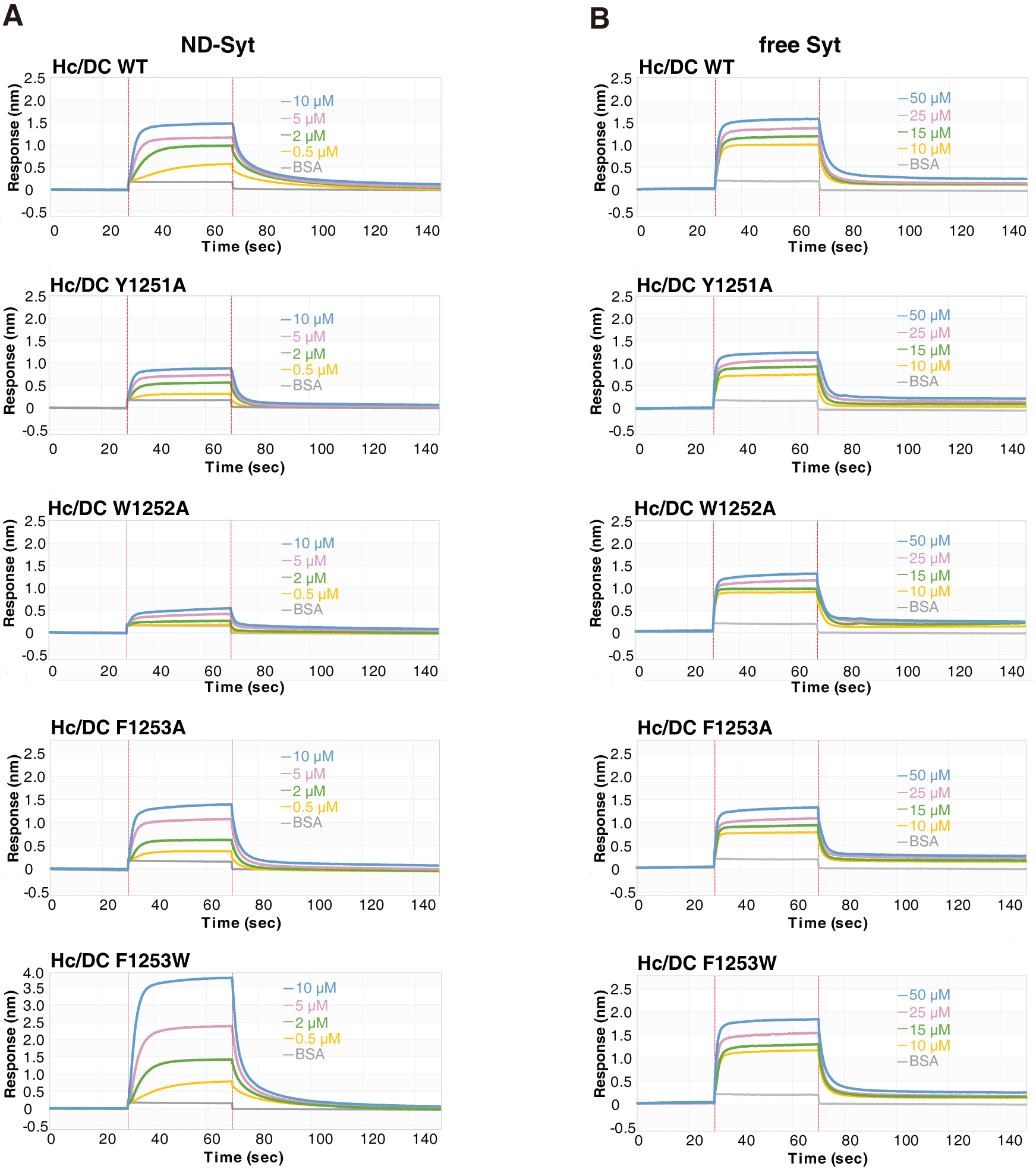

Supplement: S3 Fig — (A) Binding of WT HC/DC and the indicated HC/DC mutants to ND-Syt was analyzed by BLI assays. Binding kinetics were analyzed and are listed in Table 1. (B) Binding of WT HC/DC and the indicated HC/DC mutants to immobilized GST-tagged Syt II (1–61) protein was analyzed by BLI assays. The binding kinetics were analyzed and listed in Table 1. Mutating the tip residues in HC/DC-LBL does not alter its binding to Syt II. BLI, biolayer interferometry; GST, glutathione S-transferase; HC, C-terminal receptor-binding domain; LBL, lipid-binding loop; ND, nanodisc; Syt, synaptotagmin; WT, wild-type. (TIF) [file pbio.3000618.s003.tif]

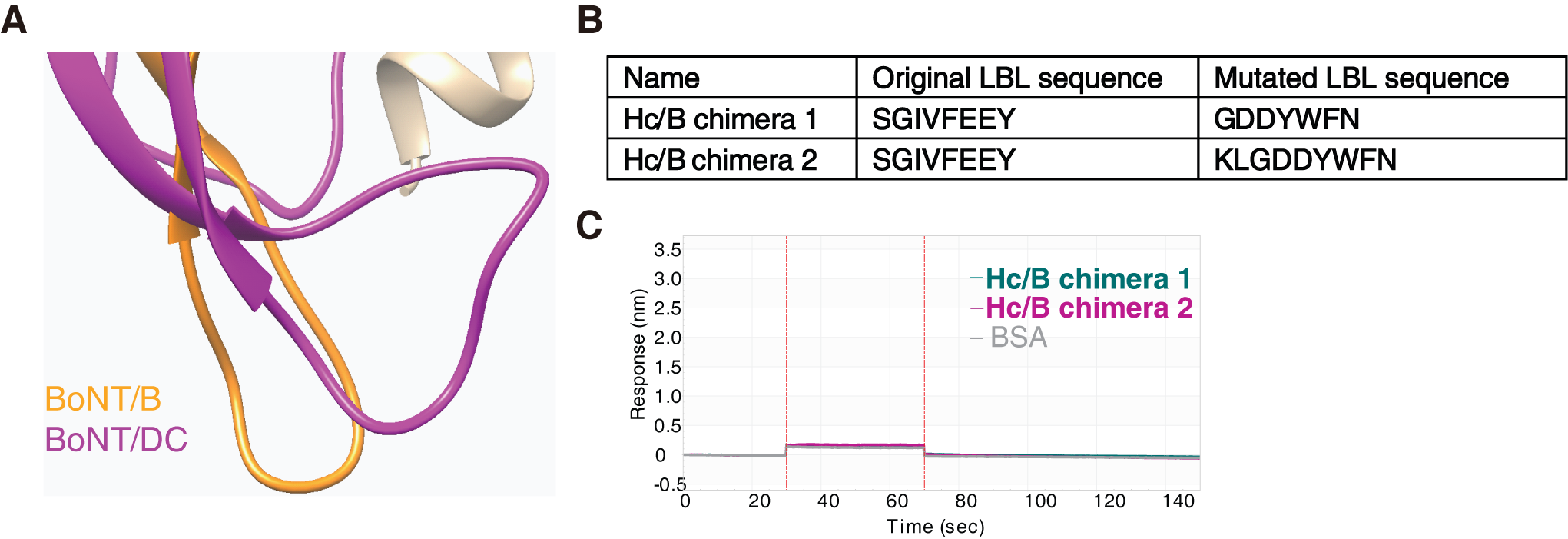

Supplement: S4 Fig — (A) Superposition of the LBLs of HC/DC and HC/B. (B) Sequence changes in HC/B. (C) The indicated HC/B mutants were examined for binding to ND1 using BLI at 10 μM concentrations. BLI, biolayer interferometry; HC, C-terminal receptor-binding domain; LBL, lipid-binding loop; ND1, receptor-free nanodisc. (TIF) [file pbio.3000618.s004.tif]

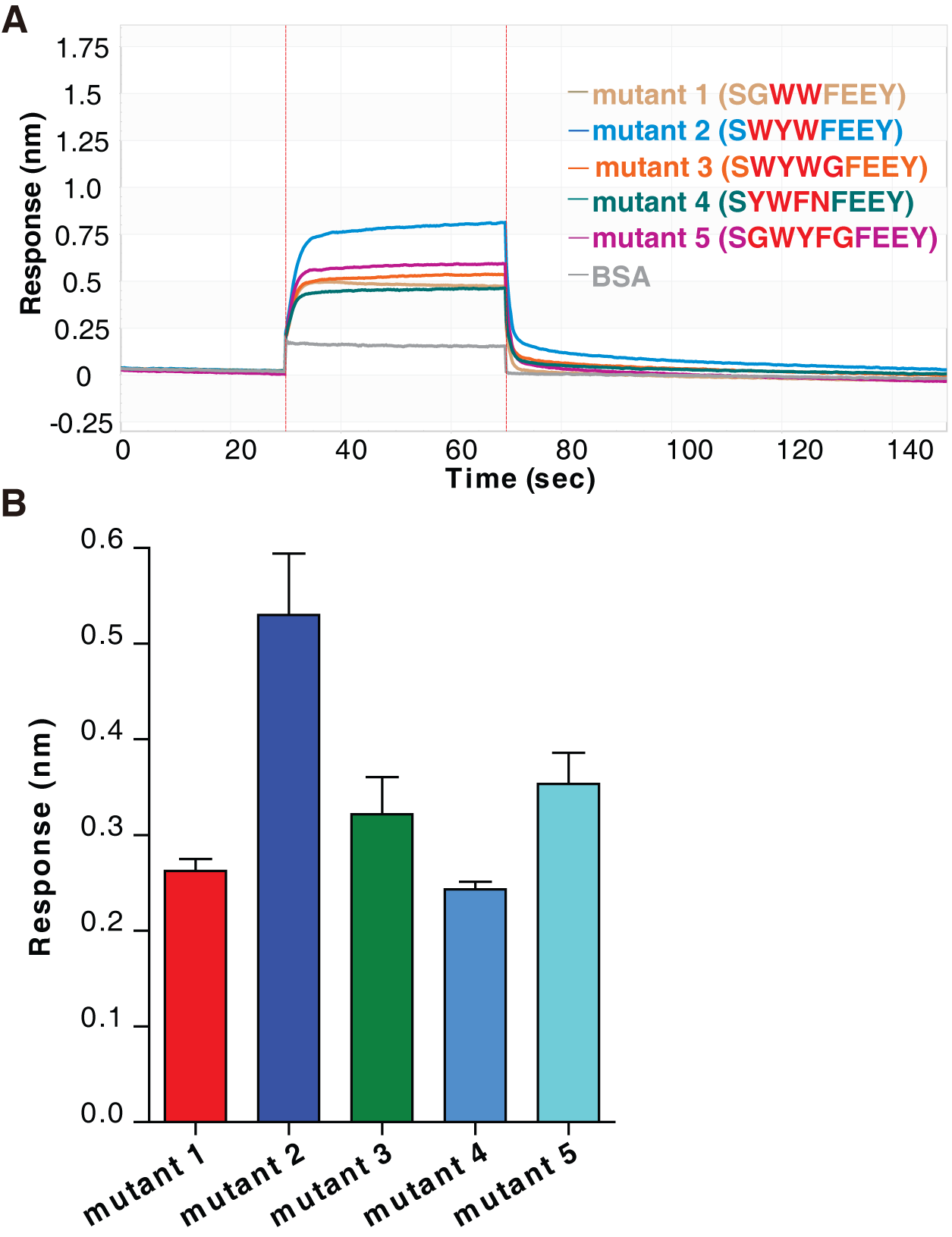

Supplement: S5 Fig — (A) Binding of the indicated mutants to ND1 was analyzed using BLI at 10 μM concentration. The specific mutations in LBL are marked in red. (B) The maximal binding signals of HC/B mutants (10 μM) to ND1 were plotted in the bar graph. Error bars indicate means ± SD, n = 3. Numerical values for (B) are available in S1 Data. BLI, biolayer interferometry; HC, C-terminal receptor-binding domain; LBL, lipid-binding loop; ND1, receptor-free nanodisc. (TIF) [file pbio.3000618.s005.tif]

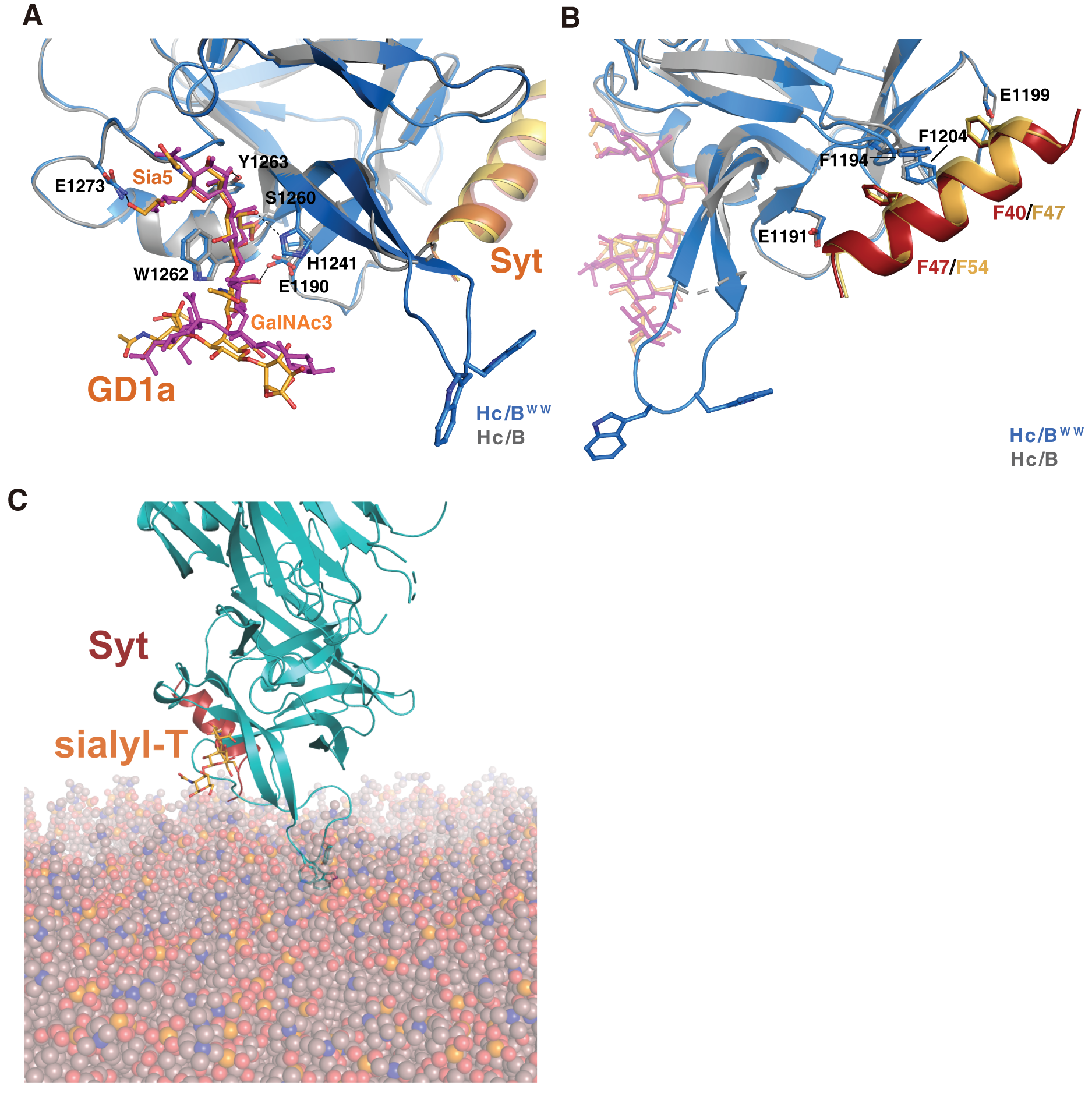

Supplement: S6 Fig — (A) The ganglioside-binding site. GD1a in the WT costructure in pink, and blue in HC/BWW complex. Main residues involved in binding are shown as sticks, with hydrogen bonds represented as dashed lines. (B) The Syt-binding site. Residues involved in binding are highlighted, illustrating the high similarity between the mutant and WT HC/B bound to hSyt I (red) and rSyt II (yellow), respectively. (C) The structures of HC/DC (cyan) in complex with hSytI (PDB 4isq) and the sialyl-T carbohydrate (PDB 5lr0) shows that the LBL loop (residues 1251–1253) interacts with the membrane. GD1a, disialoganglioside; HC, C-terminal receptor-binding domain; hSyt I, human Syt I; LBL, lipid-binding loop; PDB, Protein Data Bank; rSyt II, rat Syt II; Syt, synaptotagmin; WT, wild-type. (TIF) [file pbio.3000618.s006.tif]

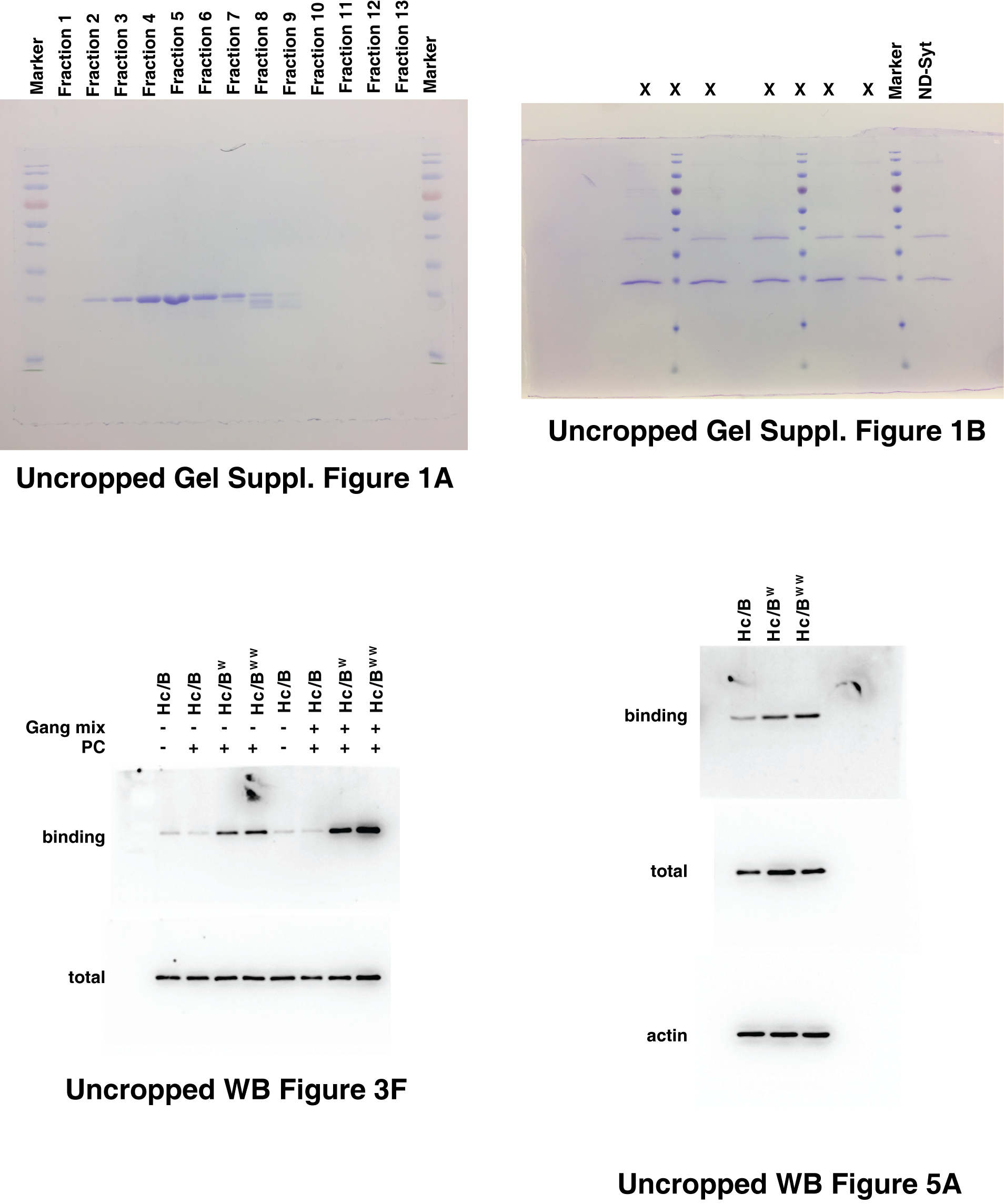

Supplement: S1 Raw Images — (TIF) [file pbio.3000618.s010.tif]
