# Supplementary material for: Characterization of a membrane binding loop leads to engineering botulinum neurotoxin B with improved therapeutic efficacy
Source: PLoS Biol. 2020 Mar 17;18(3):e3000618. doi: 10.1371/journal.pbio.3000618 (PMC7077807; doi:10.1371/journal.pbio.3000618)
Supplement: S1 Table — HC, C-terminal receptor-binding domain; WT, wild-type. (DOCX) [file pbio.3000618.s007.docx]

**S1 Table. Kinetics and affinity analysis of WT H_C_/B and H_C_/B^WW^ binding to nanodiscs.**

| Ligand | Analyte | KD (M) | ka (1/Ms) | ka Error | kd (1/s) | kd Error |
| --- | --- | --- | --- | --- | --- | --- |
| ND-CGs | H_C_/B | 5.1E-05 | 5.3E+03 | 9.5E+02 | 2.7E-01 | 1.6E-02 |
|  | H_C_/B^WW^ | 1.2E-05 | 6.0E+03 | 1.5E+03 | 7.3E-02 | 1.3E-03 |
| ND-Syt | H_C_/B | 8.8E-08 | 9.3E+04 | 5.8E+02 | 8.2E-03 | 1.5E-05 |
|  | H_C_/B^WW^ | 1.1E-07 | 7.7E+04 | 3.4E+02 | 8.4E-03 | 9.7E-06 |
| ND-Syt-CGs | H_C_/B | 6.4E-09 | 1.7E+05 | 2.8E+02 | 1.1E-03 | 1.7E-06 |
|  | H_C_/B^WW^ | <4.1E-09 | 2.4E+05 | 1.2E+03 | <9.8E-04 | 4.8E-06 |
