# Supplementary material for: Characterization of a membrane binding loop leads to engineering botulinum neurotoxin B with improved therapeutic efficacy
Source: PLoS Biol. 2020 Mar 17;18(3):e3000618. doi: 10.1371/journal.pbio.3000618 (PMC7077807; doi:10.1371/journal.pbio.3000618)
Supplement: S2 Table — (DOCX) [file pbio.3000618.s008.docx]

**S2 Table. X-ray crystallography: data collection and refinement statistics**

|  | **H_C_B^WW^:hSyt I:GD1a** |
| --- | --- |
| **Data collection** |  |
| Space group | *P2_1_2_1_2_1_* |
| Cell dimensions |  |
| *a*, *b*, *c* (Å) | 50.4, 78.5, 149.3 |
| α, β, γ (°) | 90.0, 90.0, 90.0 |
| Resolution (Å) | 2.4-74.5 (2.40-2.46)* |
| No. total/unique reflections | 248,236 / 23,939 |
| *R*_merge_ | 0.117 (14.7) |
| *R*_pim_ | 0.054 (0.676) |
| CC_1/2_ | 0.998 (0.907)* |
| *I* / σ*I* | 9.9 (1.2)* |
| Completeness (%) | 100 (100)* |
| Redundancy | 10.4 (10.5)* |
|  |  |
| **Refinement** |  |
|  |  |
| *R*_work_ / *R*_free_ | 20.1 / 22.9 |
| No. atoms |  |
| Protein / Peptide | 3,676 / 137 |
| GD1a oligosaccharide | 88 |
| Water | 85 |
| *B*-factors |  |
| Protein / Peptide | 69.4 / 89.9 |
| GD1a oligosaccharide | 101.7 |
| Water | 54.4 |
| R.m.s. deviations |  |
| Bond lengths (Å) | 0.008 |
| Bond angles (°) | 1.54 |
| Ramachandran statistics (Molprobity) |  |
| Favored (%) | 95.7 |
| Outliers (%) | 0.0 |

*Values in parentheses are for highest-resolution shell.
